# The *PROSCOOP10* Gene Encodes Two Extracellular Hydroxylated Peptides and Impacts Flowering Time in Arabidopsis

**DOI:** 10.3390/plants11243554

**Published:** 2022-12-16

**Authors:** Marie-Charlotte Guillou, Thierry Balliau, Emilie Vergne, Hervé Canut, Josiane Chourré, Claudia Herrera-León, Francisco Ramos-Martín, Masoud Ahmadi-Afzadi, Nicola D’Amelio, Eric Ruelland, Michel Zivy, Jean-Pierre Renou, Elisabeth Jamet, Sébastien Aubourg

**Affiliations:** 1 Institut Agro, SFR QUASAV, IRHS, Université Angers, INRAE, F-49000 Angers, France; 2 AgroParisTech, GQE—Le Moulon, PAPPSO, Université Paris-Saclay, INRAE, CNRS, F-91190 Gif-sur-Yvette, France; 3 Laboratoire de Recherche en Sciences Végétales, Université de Toulouse, UPS, Toulouse INP, CNRS, F-31320 Auzeville-Tolosane, France; 4 Unité de Génie Enzymatique et Cellulaire UMR 7025 CNRS, Université de Picardie Jules Verne, F-80039 Amiens, France; 5 Department of Biotechnology, Institute of Science and High Technology and Environmental Sciences, Graduate University of Advanced Technology, Kerman 117-76315, Iran; 6 Unité de Génie Enzymatique et Cellulaire UMR 7025 CNRS, Université de Technologie de Compiègne, F-60203 Compiègne, France

**Keywords:** phytocytokine, apoplasm, post-translational modification, flowering, Arabidopsis, peptidomics

## Abstract

The Arabidopsis *PROSCOOP* genes belong to a family predicted to encode secreted pro-peptides, which undergo maturation steps to produce peptides named SCOOP. Some of them are involved in defence signalling through their perception by a receptor complex including MIK2, BAK1 and BKK1. Here, we focused on the *PROSCOOP10* gene, which is highly and constitutively expressed in aerial organs. The MS/MS analyses of leaf apoplastic fluids allowed the identification of two distinct peptides (named SCOOP10#1 and SCOOP10#2) covering two different regions of PROSCOOP10. They both possess the canonical S-X-S family motif and have hydroxylated prolines. This identification in apoplastic fluids confirms the biological reality of SCOOP peptides for the first time. NMR and molecular dynamics studies showed that the SCOOP10 peptides, although largely unstructured in solution, tend to assume a hairpin-like fold, exposing the two serine residues previously identified as essential for the peptide activity. Furthermore, *PROSCOOP10* mutations led to an early-flowering phenotype and increased expression of the floral integrators *SOC1* and *LEAFY*, consistent with the de-regulated transcription of *PROSCOOP10* in several other mutants displaying early- or late-flowering phenotypes. These results suggest a role for *PROSCOOP10* in flowering time, highlighting the functional diversity within the *PROSCOOP* family.

## 1. Introduction

Small secreted peptides originate from the processing of protein precursors that share an N-terminal signal peptide directing them to the secretory pathway. We can distinguish between (i) small post-translationally modified peptides (PTMPs) produced by proteolytic processing [1] and (ii) cysteine-rich peptides (CRPs) characterised by an even number of cysteine residues involved in intramolecular disulphide bonds [2,3]. An integrative approach combining bioinformatics, transcriptomics and phenotyping resulted in the identification of a gene family encoding precursors of putative small PTMPs named serine-rich endogenous peptides (SCOOPs) [4]. All the predicted SCOOP peptides share an S-X-S motif and are specific to the *Brassicaceae* species. Moreover, assays based on application of synthetic peptides have shown that SCOOP peptides are phytocytokines involved in defence signalling through their perception by the MDIS1-interacting leucine-rich repeat receptor kinase 2 (MIK2) [5,6] and the two co-receptors BRI1-associated receptor kinase (BAK1) and BAK1-LIKE 1 (BKK1) [4,5,6]. New studies tolerating greater variability in the C-terminal motif of the PROSCOOP amino acid sequence have expanded the SCOOP family to 28 members [7]. For example, the SECRETED TRANSMEMBRANE PEPTIDE family (STMP) contains ten members, out of which four have also been annotated as SCOOP peptides: STMP1, STMP2, STMP8 and STMP10 actually correspond to SCOOP13, SCOOP14, SCOOP15 and SCOOP4, respectively [8]. Additionally, the ENHANCER OF VASCULAR WILT RESISTANCE 1 peptide (EWR1) and four closely related peptides also encode functional SCOOP peptides [7]. All these peptides share SCOOP characteristics and can induce MIK2-dependent immune responses. However, the transcription profiles of the *PROSCOOP* genes differ according to organs and various stimuli. This raises the question of the involvement of the SCOOP peptides in different biological functions; notably, in plant development. Indeed, *PROSCOOP12* (*AT5G44585*) is constitutively expressed in roots and recent studies have shown that SCOOP12 moderates root elongation through the control of reactive oxygen species (ROS) homeostasis [9]. This study focuses on *PROSCOOP10* (*AT5G44580*), which is highly expressed in aerial parts [10,11]. We show that *PROSCOOP10* encodes a propeptide, which gives rise to two distinct peptides that we identified in leaf apoplastic fluids. We identified a tendency towards hairpin structures in these peptides exposing the S-X-S SCOOP motif. We also found an early-flowering phenotype in *proscoop10* mutants, suggesting a role in the development of the aerial part of the plant, particularly in flowering time.

## 2. Results

### 2.1. Identification of SCOOP10 Peptides in Extracellular Fluids by MS

**Figure 1:**
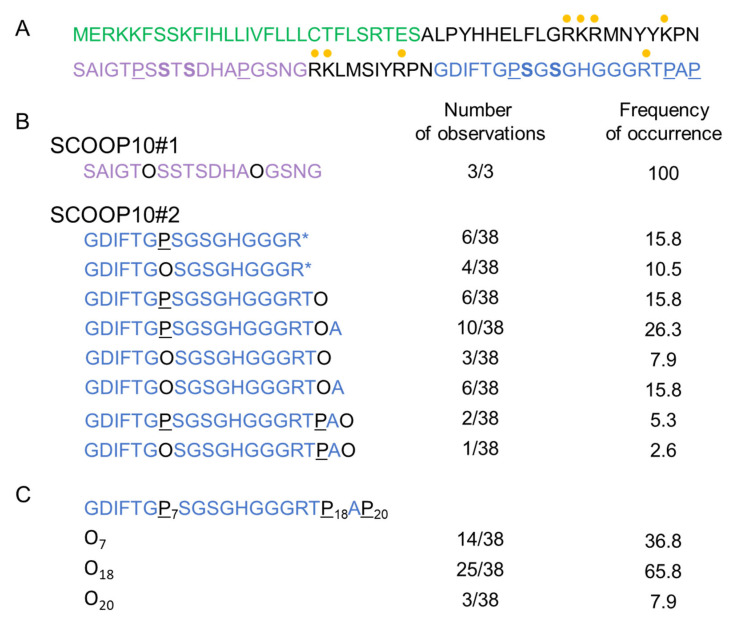
Identification of two SCOOP10 peptides by MS. (**A**) Sequence of the prepropeptide PROSCOOP10. The predicted signal peptide is in green, the mature SCOOP10#1 in purple and the mature SCOOP10#2 in blue. The tryptic cut sites (arginine (R) and lysine (K)) are indicated with yellow dots. The proline residues that were found to be hydroxylated are in black and underlined (P). The conserved serine residues are in bold. (**B**) Description of the different peptides covering the SCOOP10#1 and SCOOP10#2 amino acid sequences. The positions of P hydroxylation are indicated with O, which stands for hydroxyproline. Stars indicate that the peptide was identified after tryptic digestion. The frequency of observations of each peptide is indicated, as well as the percentage of occurrences. (**C**) A focus on SCOOP10#2 to show the number of observations and the frequency of hydroxylation events at each P position.

Based on the high expression level of *PROSCOOP10* in leaves, we used a proteomic approach to explore the apoplastic fluid content and search for the native form(s) of SCOOP10. The experiment was repeated thrice: twice without mannitol in the infiltration buffer and once in the presence of 0.3 M mannitol. The results were similar. In one case, tryptic digestion of the proteins was performed prior to the MS analysis. Different peptides could be identified that covered two different regions of PROSCOOP10: they are indicated as SCOOP10#1 and SCOOP10#2 in Figure 1. The corresponding MS/MS spectra are shown in Supplementary Figure S2. The native SCOOP10#1 peptide comprises 18 amino acids and covers the central predicted conserved motif defined after the comparison of the amino acid sequences of the members of the PROSCOOP family [4]: SAIGTPSSTSDHAPGSNG (Figure 1A,B). It contains the two strictly conserved serine (S) residues. All the observed peptides were native ones, as shown by the absence of tryptic sites at their N- and C-termini. SCOOP10#1 was only observed thrice compared to the 38 observations of SCOOP10#2. In all these three occurrences, it contained two hydroxyproline (O) residues. The native SCOOP10#2 peptide comprises 20 amino acids at the most and covers the C-terminal predicted conserved motif of the PROSCOOP family [4]: GDIFTGPSGSGHGGGRTPAP (Figure 1A,C). As with SCOOP10#1, it also contains the two strictly conserved serine residues. It further carries three well-conserved successive glycine (G) residues. The C-terminus observed at arginine (R)16 in ten cases resulted from tryptic digestion, and the corresponding peptides were not found in the absence of tryptic digestion. Thus, they should not be considered as native ones. All the other observed peptides were native ones because they were not surrounded by tryptic sites. In contrast to SCOOP10#1, the pattern of proline (P) hydroxylation was variable: we could observe hydroxyproline residues at any of the three possible positions (P7, P18 or P20) or in various combinations (Figure 1B). The most frequently observed position for P hydroxylation was P18 (65.8%), followed by P7 (36.8%). In some cases, P hydroxylation was observed at two positions on the same peptide: P7 and P18 (23.7%) or P7 and P20 (7.9%). The C-terminus of the peptide was variable, ending with P18, alanine A19 or P20. Although protease inhibitors were used already at the beginning of the experiment, the variability in the C-terminus could have been due to proteolysis by serine carboxypeptidases identified in cell wall proteomes (see *WallProtDB-2* [12,13]).

### 2.2. SCOOP10#2 Is Unstructured in Solution but Cis-Trans Isomerization May Be Favoured in a Hydrophobic Environment

According to MS analyses, SCOOP10#2 seems to be the major secreted, or the more stable, peptide produced by *PROSCOOP10*. Therefore, now knowing its exact sequence, we decided to study its structural behaviour in solution using a hydroxylated synthetic SCOOP10#2 peptide (GDIFTGOSGSGHGGGRTOAP) and a non-hydroxylated one, SCOOP10#2* (GDIFTGPSGSGHGGGRTPAP).

**Figure 2:**
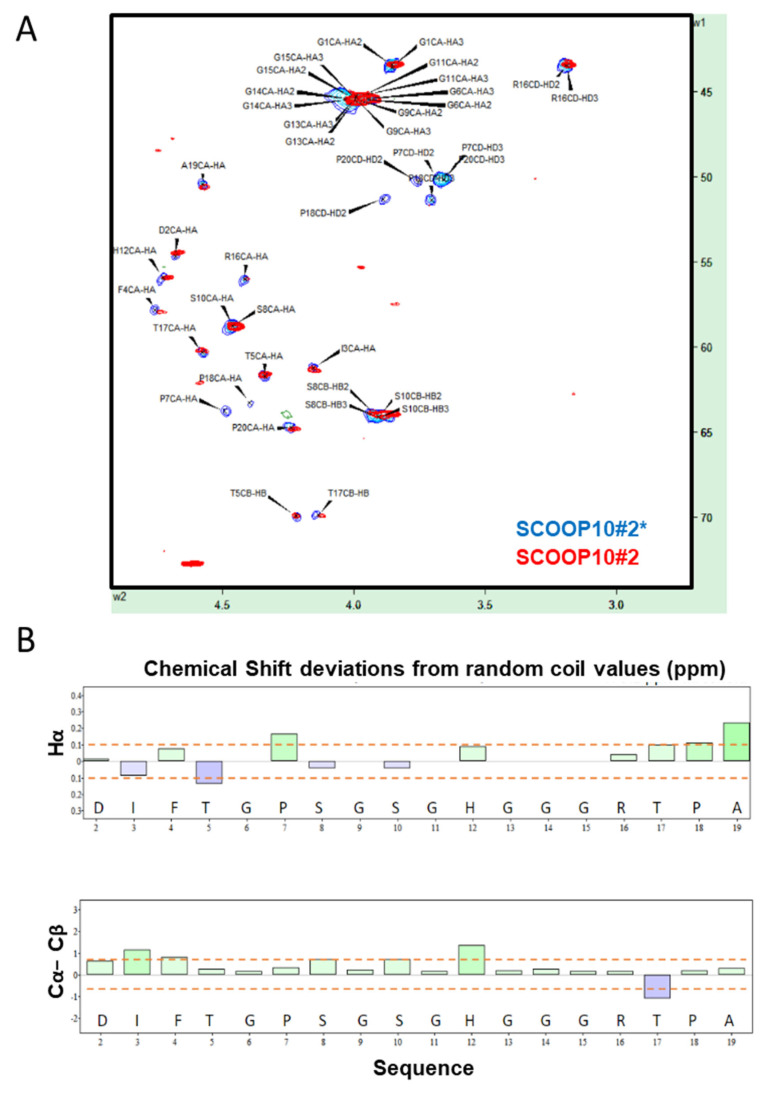
Structural behaviour of SCOOP10#2 and SCOOP10#2* in solution. (**A**) ^1^H,^13^C—HSQC spectrum assignment of non-hydroxylated SCOOP10#2* (blue) and hydroxylated SCOOP10#2 (red); 0.5 mM in 50 mM phosphate buffer at pH 6.6 and 278 K. (**B**) Chemical shift deviations from random coil values for Hα protons and for the difference between Cα and Cβ carbons suggest the absence of a well-defined structure for SCOOP10#2. Deviations for glycine Hα atoms were intentionally omitted.

NMR data indicated that the synthetic SCOOP10#2 peptide was largely unstructured in water solution. The NMR assignments of both non-hydroxylated (SCOOP10#2*) and hydroxylated (SCOOP10#2) forms are reported in Supplementary Tables S2 and S3, respectively. The chemical shift of various nuclei—namely, Hα, Cα, Cβ and carbonyl—can be used to ascertain the presence of a secondary structure by observing their deviations from random coil values [14,15,16], which can be predicted by the peptide sequence [17]. The Hα/Cα region of the ^1^H,^13^C-HSQC NMR spectrum is shown in Figure 2A, while the chemical shift deviations from random coil values are reported in Figure 2B. The presence of alpha helical structure can be monitored using at least three consecutive negative Hα or Cβ or positive Cα deviations, while the opposite holds for beta strands. The difference between Cα and Cβ deviations is often used as a combined predictor. Overall, the observed deviations were below the threshold value of 0.1 ppm for ^1^H and 0.7 ppm for ^13^C [18]. Hydroxylation of P7 and P18 had no major impact on the structure, as indicated by the minimal chemical shift perturbations at most resonances (Figure 2A). Large changes were limited to the atoms of hydroxyprolines (O) and, particularly, to Cγ carbon atoms, as expected.

NMR spectra also allowed the monitoring of the conformation of the peptide bond linking proline residues to the previous amino acid [19,20]. Such a bond can often assume a *cis* conformation, whereas it is commonly found as *trans* in all other amino acid types [21]. A *cis* conformation determines a rather radical change in the direction of the peptide backbone and might be crucial for the biological function. In particular, the Hα of the preceding residue is close in space to the Hα or Hδ protons of proline in the *cis* and *trans* conformations, respectively. Its resonance can therefore be used to identify each conformer in NOESY or ROESY spectra [22]. A clear NOESY peak between the Hα protons of G6 and Hδ protons of P7 (or O7) revealed that the peptide bond was mainly in the *trans* conformation.

In order to investigate the structural behaviour of SCOOP10#2 in a more apolar environment that could mimic that of its receptor, we studied the peptide in DMSO (Supplementary Figure S3 and Supplementary Tables S4 and S5). Interestingly, we observed a doubling of peaks arising from residues 6–10, which is the region comprising the two conserved serine residues (Figure 1A). Analysis of NOESY spectra revealed that the minor form might belong to the *cis* conformation of the G6-P7 peptide bond, as a cross peak was present between the Hα proton of G6 and an Hα compatible with the second form of P7. The same was not observed for P18 and P20.

### 2.3. Transient Hairpin-like Structures Exposing S8 and S10 Might Have Functional Relevance

**Figure 3:**
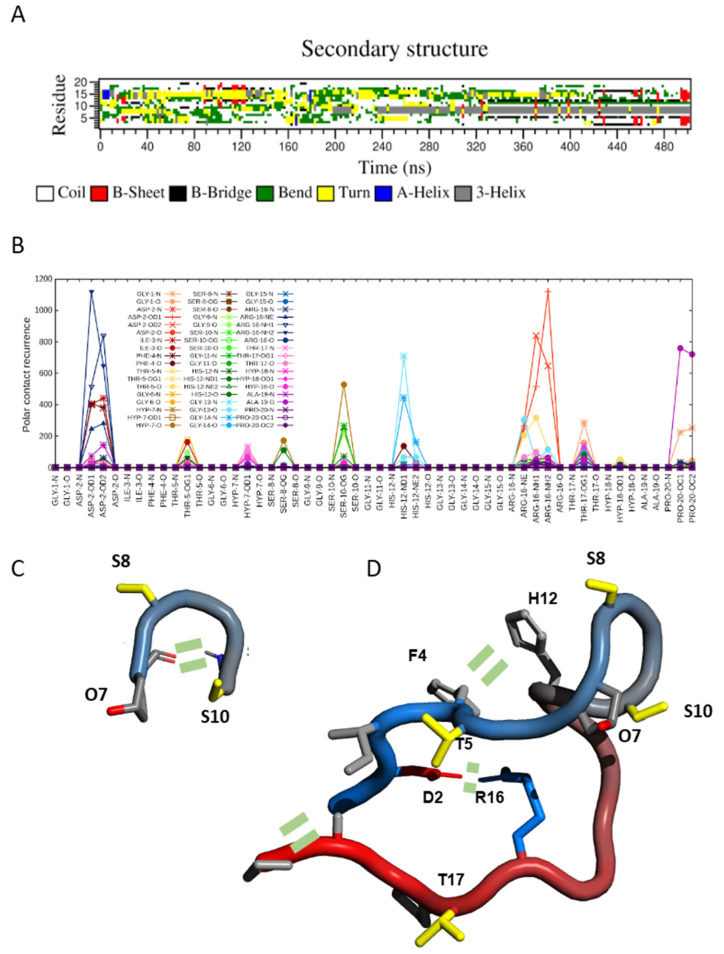
Secondary structures and intramolecular interactions found in MD simulations of SCOOP10#2. (**A**) DSSP secondary structures calculated in molecular dynamics (MD) simulation of hydroxylated SCOOP10#2 in solution. (**B**) Occurrence of intramolecular polar atom contacts (H-bonds and salt bridges) in SCOOP10#2 calculated in MD simulation trajectories. (**C**,**D**) Schematic representations of SCOOP10#2 shown as a “tube” coloured from blue (N-terminus) to red (C-terminus). Side-chains are shown as sticks with the following colour code: positively charged (blue) and non-polar (light grey). The structures were created with PyMol [23]. Key intramolecular interactions are indicated by two short parallel dashes.

Molecular dynamics simulations also pointed to the absence of a well-defined structure in both SCOOP10#2* and its hydroxylated form, in agreement with the NMR data. Indeed, during MD simulations, an ensemble of different conformations continuously interconverted along the trajectory (Figure 3A). However, these data revealed a certain tendency for the backbone to fold at the level of residues O7-S10, thus exposing S8 and S10 side chains. Interestingly, this region was the one displaying negative Hα and positive Cα-Cβ deviations in the NMR, as expected for the turn of a helix (Figure 3B). Although below the threshold values, these data suggest the presence of a small population of conformers contributing to the average chemical shift value. For this reason, we analysed molecular dynamic trajectories in order to reveal key interatomic interactions that might stabilise such a fold. For SCOOP10#2, the obtained polar contacts map revealed the formation of an H-bond between the amide nitrogen of serine S10 and the carbonyl of hydroxyproline O7 (Figure 3C), a salt bridge between the side chains of aspartic acid D2 and arginine R16 and a salt bridge between the C- and N-termini (Figure 3D). Furthermore, stacking between the aromatic rings of phenylalanine F4 and histidine H12 might also have further stabilised the conformation (Figure 3D).

In order to test the relative contribution of each of these key inter-residue interactions, we performed multiple molecular dynamic simulations where we mutated at least one partner residues: R16A, G13,14,15A, P7A and S8,10A (Supplementary Figure S4A). In the R16A mutant, where we eliminated the salt bridge between D2 and R16, SCOOP10#2* still tended to fold onto itself but at the level of residues 12–15 (Supplementary Figure S4B), thus reducing the exposition of S8 (Supplementary Figure S5). In this case, the driving force was the salt bridge between the N and C termini. When glycine residues in the flexible region G13,14,15 were mutated to alanine, the N–C terminal interaction favoured a fold in the middle of the structure (residues 9–10) (Supplementary Figure S4B). As for the H-bond between the amide of S10 and P7, mutants (P7A and double mutant S8,10A) revealed small perturbations in the structural behaviour (in P7A, the structure folded around residue 8 in a turn rather than a helix as in residues 3–10), an effect expected considering that the interaction was established at the level of the backbone.

### 2.4. SCOOP10#1 Is Unstructured in Solution with Transient Head-to-Tail Contact

We also studied the structural behaviour in solution of SCOOP10#1 using a synthetic SCOOP10#1 peptide identical to the native form identified (SAIGTOSSTSDHAOGSNG). For the synthetic SCOOP10#1 peptide, deviations from random coil values also indicated poor structuring (see Supplementary Figure S6 and the NMR assignment in different conditions in Supplementary Tables S6 and S7), and NOESY spectra were compatible with the *trans* conformation for the peptide bonds involving both P6 and P14. In contrast to what was observed for SCOOP10#2, we could not detect the presence of the *cis* conformation in the more apolar environment of DMSO. MD simulations (Supplementary Figure S7) agreed with the NMR data and detected a lack of structure (Supplementary Figure S7A); however, infrequent contacts between the N- and C-termini (Supplementary Figure S7B) generated folded conformations vaguely resembling those observed for SCOOP10#2 (see contact map in Supplementary Figure S7C).

### 2.5. Native SCOOP10 Peptides Do Not Induce Strong ROS Production and Growth Inhibition

**Figure 4:**
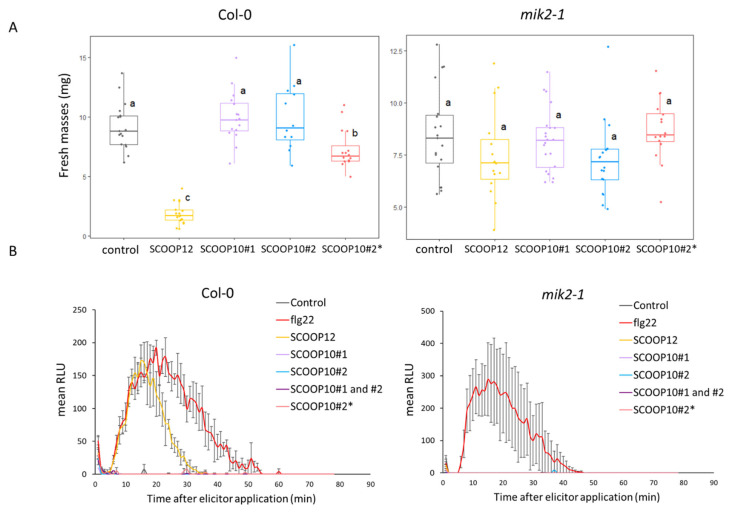
Effects of SCOOP10 peptides on seedling growth and on ROS production. (**A**) Seedling growth inhibition evaluation by measuring fresh mass after 1 µM elicitor or control treatment. (**B**) H_2_O_2_ production after 1 µM elicitor or control treatment measured with a luminol-based assay using leaf discs from four-week-old plants of the indicated genotypes. Data represented are means of three independent replicates in relative luminescence units (RLUs) (*n* = 3, ±SEM). For (**A**,**B**), SCOOP10#1 and SCOOP10#2 correspond to the proline hydroxylated peptides, and prolines are not hydroxylated for the SCOOP10#2*peptide. ANOVA and Tukey test results allow us to define significantly different groups, labelled from a to c, at the *p* < 0.05 level.

Based on their identified native sequences, we tested the effects of the SCOOP10 synthetic peptides on seedling growth and ROS production in Col-0 and *mik2-1* genotypes in comparison to the effects of the SCOOP12 and flg22 peptides as positive controls (Figure 4). The SCOOP12 amino acid sequence was the same as that already used by Gully et al. [4] and Guillou et al. [9] and based on the prediction without post-translational modifications.

In the Col-0 background and at 1 µM, we showed that the SCOOP10#2* peptide induced seedling growth inhibition that was much smaller than that induced by the SCOOP12 peptide. In the same conditions, the SCOOP10#1 and SCOOP10#2 peptides did not show significant effects. In the *mik2-1* mutant background, none of the SCOOP peptides had an effect on growth, as expected (Figure 4A). At the same concentration, the SCOOP12 peptide induced strong ROS production in Col-0 leaves, as already reported [4,6]. In contrast, neither the SCOOP10#2* nor the SCOOP10#1 and SCOOP10#2 peptides, even when simultaneously applied, induced ROS production in leaves. As expected, none of the SCOOP peptides induced high ROS production in the *mik2-1* mutant (Figure 4B).

### 2.6. Mutations of PROSCOOP10 Impact Flowering Time

Previous analysis of transcriptomic data showed that *PROSCOOP10* is highly expressed in shoot apexes and leaves and may play a role in aerial organ development [4,5]. Therefore, we compared the leaf development and flowering time of wild-type and *proscoop10* plants. The first *proscoop10* mutant (*proscoop10-1*) was a T-DNA insertion line ordered from NASC (Supplementary Figure S1A). This line was genotyped as mutated on both DNA and cDNA (Supplementary Figure S1B). We generated a second line (*proscoop10-2*) using a CRISPR/Cas9 approach and the mutant was genotyped (Supplementary Figure S1C,D). *proscoop10-2* was a bi-allelic mutant, which are frequently observed with this approach [23]. In the first modified allele, a deletion of 237 bp occurred between the two guides in addition to modifications of nucleotides within the guides, leading to a frameshift. For the second modified allele, a deletion of 448 bp occurred between the two guides (Supplementary Figure S1E). In each case, the modifications prevented the synthesis of the native SCOOP10 peptides (Supplementary Figure S1F).

**Figure 5:**
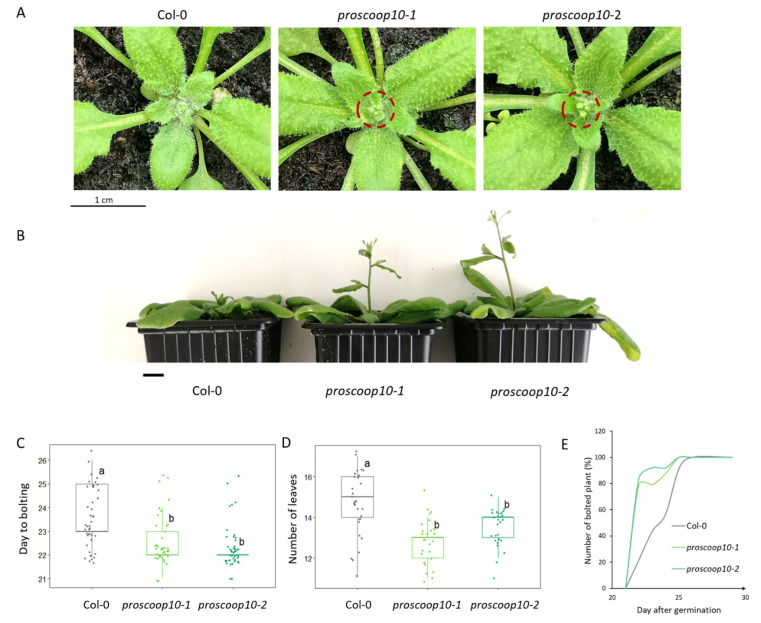
*proscoop10* early-flowering phenotype. (**A**) Bolted inflorescence of the *proscoop10* mutant lines, 23nd day after germination. Red circles highlight the bolted inflorescences of the *proscoop10* mutant lines compared to Col-0 on the same day. Scale bar = 1 cm. (**B**) Delay in stem development between mutants and WT due to the early flowering of the *proscoop10* lines, 27th day after germination. Scale bar = 1 cm. (**C**) Average day to bolting for each genotype (*n* > 40). (**D**) Average number of rosette leaves at the flowering time for each genotype (*n* > 25). For (**C**,**D**), ANOVA and Tukey tests made it possible to significantly define two different groups labelled a and b. (**E**) Kinetics represent the numbers of bolted inflorescences in %, starting from day 20 to day 30 after germination (*n* = 25).

Both mutant lines displayed normal vegetative development but, 22 days after germination, the inflorescences of *proscoop10* mutants started to bolt significantly earlier than those of Col-0 plants, with a shift of two days (Figure 5A,C), and the numbers of rosette leaves were significantly lower for *proscoop10* at the bolting day (Figure 5D). Consequently, 27 days after germination, mutants had longer stem length compared to Col-0 (Figure 5B). Although the delay in flowering between mutant and Col-0 plants was slight, 80% of the inflorescences of the *proscoop10* mutants bolted around the 22nd day after germination, whereas the inflorescence of wild-types bolted gradually from the 23rd to the 26th day after germination (Figure 5E, Supplementary Table S8).

**Figure 6:**
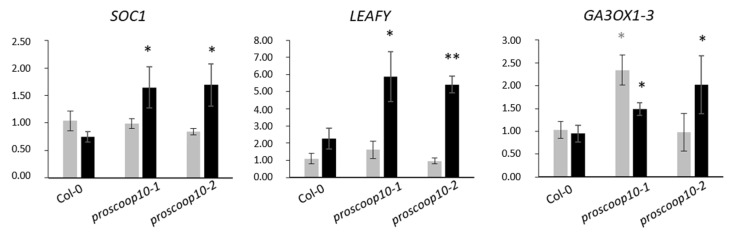
Impact of *PROSCOOP10* mutation on transcription of genes involved in floral regulation. Relative expression of *SOC1*, *LEAFY* and *GA3OX-1* genes was measured by RT-qPCR after 7 days (in grey) and 11 days (in black) in the two *proscoop10* mutant lines compared to Col-0 at 7 days. Values represent mean ratios ± SEM of three independent biological replicates. Asterisks denote statistical differences in gene expression levels between *prosccoop10* mutants and Col-0: * *p* < 0.05, ** *p* < 0.01 (ratio-paired *t*-test).

Based on the mutant phenotype, we next examined the expression of three genes involved in floral regulation, *SUPPRESSOR OF CONSTANS 1* (*SOC1)*, *LEAFY* and *GA3OX-1*, in the SAM of *proscoop10* mutants and Col-0 at 7 and 11 days after germination (Figure 6). At 7 days, the two floral transition genes, *SOC1* and *LEAFY*, were not differentially expressed between mutants and Col-0, whereas *GA3OX-1* was induced only in *proscoop10-1*. At 11 days, all three genes were up-regulated in the two *proscoop10* mutant lines compared to Col-0.

## 3. Discussion

### 3.1. Two Distinct Hydroxylated SCOOP10 Peptides Are Present in the Leaf Apoplasm

The MS analysis of apoplastic fluid samples from rosette leaves identified two hydroxylated SCOOP10 peptides (18 and 20 aa long) resulting from the processing of two distinct regions of the PROSCOOP10 protein separated by ten amino acids. This result confirms that *PROSCOOP* genes indeed encode preproproteins processed in short secreted peptides. Furthermore, the presence of hydroxyproline residues confirms that SCOOPs are PTMPs according to Matsubayashi’s classification [2]. We thus characterized two distinct SCOOP10 peptides, showing the biological reality of the previous predicted peptides SCOOP10#1 and SCOOP10#2. Note that the observed native peptides were a few amino acids longer (at both their N- and C- termini) than previously predicted. As shown by MS analyses, SCOOP10#2, corresponding to the C-terminal end of the PROSCOOP10 precursor, seems to be the major form in the leaf apoplasm. The ability of a precursor protein to be processed in different peptides has been described for a few genes belonging to the CLE, CEP and PIP families [1,24,25]. In our case, the ability of *PROSCOOP10* to produce two distinct SCOOP10 peptides probably comes from the local duplication of an exon. Indeed, in contrast to the large majority of *PROSCOOP* genes, which have two coding exons (the first one encoding the signal peptide and the second one containing the conserved SCOOP motif), *PROSCOOP10* has a third exon containing a second SCOOP motif (Supplementary Figure S1A [4]). This feature is also shared by *PROSCOOP6*, *7*, *11* and *15*, which are also probably able to encode two SCOOP peptides, even if previous assays based on exogenous application of predicted synthetic peptides showed that only the C-terminal ones had biological activity [5,6]. The identification of these native SCOOP10 peptides suggests that their maturation requires cleavage steps by still unknown endoproteases. The N-termini of both SCOOP10 peptides are located upstream of the Y[KR]PN motif (Figure 1), similar to the cleavage site in IDA precursors where P and Y residues in positions -2 and -4 relative to the cleaved bond are important for cleavage site recognition by subtilases SBT4.12, SBT4.13 and SBT5.2 [26,27]. Due to its internal position in the precursor, the release of SCOOP10#1 probably requires an additional step involving the actions of another endoprotease and/or trimming by an exoprotease. Such a complex maturation process has already been described for the maturation of the CLE19 peptide through the activity of the exoprotease Zn^2+^ carboxypeptidase SOL1 in the extracellular space [28,29].

Regarding the post-translational modifications, the SCOOP10 amino acid motifs containing the P hydroxylation sites were of three types: AP, TP and GP. AP and TP are canonical motifs for the hydroxylation of P residues described for the CEP1 peptide [30] and arabinogalactan proteins [31,32]. The GP motif was found to be hydroxylated on the P residue in CLV3, CLE2 [30] and a few other proteins (for a review, see [33]). As previously reported for other cell wall proteins, the pattern of proline hydroxylation is variable [34]. It has been assumed that this variability could contribute to the regulation of biological activity or play a role in recognition of the cleavage site(s) targeted by the endoproteases, as Royek et al. [35] demonstrated with tyrosine sulphation. At this point, our data did not allow us to address the question of the *O*-glycosylation of the hydroxyproline residues, as reported for a few CLE peptides [36,37,38] and PSY1 [39].

In our conditions, exogenous application of the synthetic SCOOP10#2 peptide, based on the native forms with or without hydroxylated prolines (GDIFTGOSGSGHGGGRTOAP or GDIFTGPSGSGHGGGRTPAP, respectively), did not show any effect on seedling growth and ROS production, in contrast to SCOOP12 and some other members of the family. Divergent results have been obtained with different synthetic peptides based on shorter predicted sequences. Rhodes et al. [6] have shown that the peptide FTGPSGSGHGGGR induced slight seedling growth inhibition and a low level of ROS at 1 µM in an MIK2-dependent manner. Hou et al. [5] have shown that the peptide PNGDIFTGPSGSGHGGGR induced both strong seedling growth inhibition and ROS production. These different results highlight the divergent effects of native and predicted peptides. We cannot exclude the possibility that other mature SCOOP10 peptides exist in the apoplast that would be produced under specific conditions; notably, to induce immune responses when needed by the plant. It is also possible that SCOOP10 has evolved to specifically regulate plant development rather than immunity. Further experiments are needed to better understand the regulation of the processing of these peptides in the different defence and developmental contexts.

### 3.2. SCOOP10 Peptides Tend to Adopt a Hairpin Structure

The mature sequence of SCOOP10 peptides being known, we addressed the question of its molecular structure. NMR revealed that both synthetic SCOOP10#1 and SCOOP10#2 appear to be mainly unstructured in solution. Molecular dynamics simulations also pointed to the absence of a well-defined structure. However, the analysis of molecular dynamics trajectories showed that the peptides transiently adopt a hairpin conformation, especially in the case of SCOOP10#2. As often observed in ligand/receptor interactions, the active form of the protein may be scarcely populated and become major only in the presence of its target [40,41]. SCOOP10#2 transient structures would be stabilized by two salt bridges. The first, between D2 and R16 side chains, favours the formation of a turn (residues 8–9) exposing S8 and S10, while the second is between the N-terminal amine and the C-terminal carboxylate. Despite the fact that the turn is not in the middle of the structure, the two salt bridges can co-exist because the three glycine residues in positions 13–15 provide backbone flexibility. This stretch of glycines, located at the C-terminus of the S-X-S motif, is a feature shared by the majority of SCOOP peptides and leads us to think that SCOOPs could adopt such a preferred conformation for the interaction with their receptor. Interestingly, this hairpin structure exposes the two conserved serine residues that define the SCOOP family and that were shown to be essential for the peptide function. Indeed, mutation of one of the two residues is fatal for SCOOP12 perception [4].

### 3.3. PROSCOOP10 Delays the Floral Transition

The mutation of *PROSCOOP10* showed an early-flowering phenotype and a lower number of leaves at bolting day compared to Col-0 plants. This observation indicates the involvement of the *PROSCOOP10* gene in flowering-time control. Multiple factors alter the flowering time, such as the photoperiod, the vernalization and the gibberellins (GAs). The pathways dependent on these factors regulate a common set of key floral integrators [42,43,44,45]. Among them, we tested the expression of the two major genes *SOC1* and *LEAFY* [42,46] and of *GA3OX1-3*, involved in the GA biosynthetic pathway and promoting flowering [47], at 7 and 11 days after germination; i.e., before and during floral transition. Indeed, Klepikova et al. [48] discovered a time point between 10 and 12 days after germination when the floral transition occurred. After 11 days, the floral integrator genes were up-regulated in the two early-flowering *proscoop10* mutants compared to Col-0 and to the 7 days condition. This suggests that the floral transition occurred earlier in these *proscoop10* mutants. *PROSCOOP10* could delay flowering time by repressing, directly or indirectly, the expression of *SOC1*, *LEAFY* and *GA30X1-3*. Moreover, these results can be correlated with the *PROSCOOP10* expression profile in the aerial parts of the plant. Mining of transcriptomic data available in Genevestigator and CATdb [10,49] revealed more information about the transcriptional regulation of *PROSCOOP10.* Indeed, in 2008, Moon et al. reported it as one of the only two genes significantly down-regulated in the *pif1-5* mutant while describing the involvement of *PIF1* in the optimization of the greening process through the regulation of chlorophyll synthesis. Interestingly, in 2018, Wu et al. also studied *PIF1* (called *PIL5*) and identified an early-flowering phenotype under long-day growth conditions for the *pil5-1* mutant in which *SOC1* and *LEAFY* were also up-regulated. Moreover, Klepikova et al. [48] monitored the transcriptome of the SAM to reveal a critical time point in Arabidopsis flower initiation. In their analysis, *AT5G44580* was highly transcribed in the SAM during the first 9 days after germination; then, the expression strongly decreased between 9 and 10 days, with a log2 fold change of -4.54, ranking 30 out of 968 genes down-regulated between these two stages. *PROSCOOP10* expression remained at this low level at later stages of development. This expression profile is intriguingly similar to that of *FLOWERING LOCUS* (*FLC*), another flowering regulator, and the opposite to *LEAFY*, whose expression increases during the transition to flowering [48]. While the expression of *PROSCOOP10* in leaves seems rather constitutive, its strikingly different expression profile in the SAM could fit with the early-flowering phenotype of the *proscoop10* mutants. Additionally, the transcriptomic profiles of *PROSCOOP10* in various mutants and experimental conditions always show a negative correlation between the level of *PROSCOOP10* expression and the time span before flowering (Table 1). Indeed, studies have shown that *PROSCOOP10* is repressed in plants showing an early-flowering phenotype, such as in the *abi4vtc2* and *phyABCDE* mutants, compared to their corresponding wild-type [50,51]. Furthermore, in mutants such as *vtc2*, *tcp4* and *ga1-3*, which display a late-flowering phenotype or no flowering at all, *PROSCOOP10* is induced [47,52,53]. In the *arf6-2/arf8-3* mutant, inflorescence stems elongate less than those of the wild-type, and flowers cease development as infertile closed buds with short petals. In this mutant, *PROSCOOP10* is also highly expressed in comparison with the wild-type [54]. All these results suggest that *PROSCOOP10* interferes with the floral transition process, upstream of *SOC1* and *LEAFY,* to delay flowering. Thus, *PROSCOOP10* could be another player in meristem identity as a delayer of floral transition, the actual signalling cascade remaining unknown. A putative role in the maintenance of the vegetative state can also be suggested in light of the work published by Moon et al. [55], as *PROSCOOP10* was one of the two genes co-regulated with *PIF1*, itself also reported as optimizing the greening process. The fact that *PROSCOOP10* is rather constitutively expressed in the green parts of the plant could support this hypothesis, as could its huge decrease in expression when the SAM acquires its floral identity [48].

Our experiments led to the identification of native SCOOP10 peptides and the effect of their PROSCOOP10 precursor KO mutation on the flowering time. At this point, we do not have evidence of the direct link between the SCOOP10 peptides and the flowering process. We also cannot exclude the possibility that the precursor itself might have a function independent of its encoded peptides, as has been recently shown for the pathogenesis-related precursor 1 (PR1/CAPE1) [56]. The respective functions of the PROSCOOP10 precursor and the SCOOP10 peptides in the flowering process need further investigation. This link between *PROSCOO10* and the flowering process, in addition to *PROSCOOP12* function in primary root elongation [4,10], illustrates the functional complexity of the PROSCOOP family in plant development.

## 4. Materials and Methods

### 4.1. Plant Material

*Arabidopsis thaliana* ecotype Columbia (Col-0) was used as a control. Two independent *proscoop10* mutant lines in the Col-0 background were used. A first line, named *proscoop10-1*, was a T-DNA insertion line obtained from NASC (SALK_059855C), and the primers used for genotyping are listed in Supplementary Table S1. T-DNA insertion was checked by PCR for *PROSCOOP10* and compared with PCR for *AtCOP1*, used as a PCR-positive control, in Col-0 and *proscoop10-1* mutant lines. The second line, named *proscoop10-2*, was created using the clustered regularly interspaced short palindromic repeats/CRISPR-associated protein 9 (CRISPR/Cas9) method. We searched for *PROSCOOP10*-specific single guide RNA (sgRNA) and checked possible off-target sites in the Arabidopsis Col-0 genome using the Crispor Tefor program (http://crispor.tefor.net). The 20 base long RNA guides with the following sequences were used: 5′-GACCACGCTCCAGGCAGTAA-3′ and 5′-ATCAGGCAGTGGGCATGGTG-3′ (Supplementary Figure S1A). Vectors and methods used to get the CRISPR/Cas9 constructs were as in Charrier et al. [60]. Arabidopsis transformation was applied as in Zhang et al. [61].

Plants used for mass spectrometry (MS) analysis were cultivated in soil under short-day conditions (8 h light at 22 °C/16 h dark at 21 °C, 70% relative humidity) for four weeks. Sodium and mercury vapour lights were used to provide a light intensity of 352.9 μmol.m^−2^.s^−1^. The soil-grown plants used for the ROS assay and phenotyping were grown under long-day conditions (16 h light at 22 °C/16 h dark at 21 °C, 70% relative humidity). Seedlings used for the seedling growth inhibition assay were grown in sterile environment under short-day conditions (8 h light at 22 °C/8 h dark at 21 °C, 70% relative humidity).

### 4.2. Synthetic Peptides

The peptides flg22 (QRLSTGSRINSAKDDAAGLQIA), SCOOP12 (PVRSSQSSQAGGR), SCOOP10#1 (SAIGTOSSTSDHAOGSNG) and SCOOP10#2 (GDIFTGOSGSGHGGGRTOAP) were obtained from GeneCust (Boynes, France), with O corresponding to hydroxyprolines. SCOOP10#2* (GDIFTGPSGSGHGGGRTPAP), corresponding to SCOOP10#2 without hydroxyprolines, was obtained from Eurogentec SA (Seraing, Belgium). Peptides were synthesized with a minimum purification level of 95% and diluted in water to the final concentration used for the assays. The SCOOP10#1 and SCOOP10#2 peptide sequences were identical to the native sequences identified by mass spectrometry.

### 4.3. Mass Spectrometry (MS) Analyses of Extracellular Fluids

The extracellular fluids of rosettes were obtained according to Boudart et al. [62] with slight modifications. The buffer used for the vacuum infiltration contained 5 mM sodium acetate at pH 4.6 (with or without 0.3 M mannitol) and three protease inhibitors: 1 mM AEBSF (ThermoFisher, Scientific, Rockford, IL, USA), 10 mM 1–10 phenanthroline (Sigma Aldrich Chimie SARL, Saint-Quentin-Fallavier, France) and 100 µM E64 (Sigma-Aldrich Chimie SARL, Saint-Quentin-Fallavier, France). After centrifugation of the vacuum-infiltrated rosettes at 200 *g*, the fluids were collected and submitted to an ultrafiltration using an Amicon^®^ Ultra 10 K device with 10,000 NMWL (Merck Chimie SAS, Darmstadt, Germany). The samples were then speedvac-dried prior to solubilization in 10 mM DTT and 50 mM ammonium bicarbonate, which was followed by alkylation with 50 mM iodoacetamide. The samples were directly desalted by solid phase extraction (SPE) on C18 cartridges (StrataTM-XL 8E-S043-TG, Phenomenex, Le Pecq, France) as described by Balliau et al. (2018) [63]. Alternatively, the samples were subjected to tryptic digestion inside the cartridge. The peptides were eluted with 70% acetonitrile/0.06% acetic acid prior to being speedvac-dried. They were finally resuspended in 2% acetonitrile/0.1% formic acid. The samples were analysed using MS with a Q ExactiveTM-Plus Hybrid Quadrupole-Orbitrap™ mass spectrometer (Thermo-Fisher Scientific, Waltham, MA, USA) coupled with an Eksigent NanoLC-Ultra^®^ 2D HPLC (AB SCIEXTM, Redwood City, CA, USA) as described previously [63], except for the chromatographic separation step, which was shortened to 45 min. The database search was performed as described previously [64], except for the enzymatic cleavage, which was specified as “no enzyme”. Protein inference was performed using the X!TandemPipeline [65] with the following parameters: peptide E-value smaller than 0.003, protein E-value smaller than 0.01 and one peptide per protein.

### 4.4. Nuclear Magnetic Resonance (NMR) Analyses

SCOOP synthetic peptides were dissolved at concentrations between 0.5 mM and 1 mM in both 50 mM phosphate buffer water solution, pH 6.6, containing 10% D_2_O and in DMSO-d6. Deuterated sodium TSP-d4 at a concentration of 100 µM was used as an internal reference for the chemical shift in aqueous buffers [14] . Measurements were performed at 278 K for aqueous and at 298 K for DMSO samples. Almost complete assignment of amide protons, non-exchanging protons and protonated ^13^C atoms was achieved in solution by ^1^H,^13^C-HSQC, ^1^H,^1^H-TOCSY (mixing time of 60 ms) and ^1^H,^1^H-NOESY (mixing time of 200 ms) as recorded on a 500 MHz (11.74 T) Bruker spectrometer (Bruker France, Palaiseau) equipped with a 5 mm Broadband Inverse (BBI) probe. TopSpin 4 (Bruker BioSpin) and NMRFAM-SPARKY [66] were used to process and analyse NMR data. Chemical shift deviations from random coil values were calculated using the “secondary chemical shift analysis” option of NMRFAM-SPARKY [66], a module based on PACSY [67].

### 4.5. Molecular Dynamics (MD) Simulations

The starting structures for our peptides were obtained with I-Tasser [68]. In silico mutagenesis was performed in CHIMERA using the Rotamers tool [69]. The proline residues were replaced by hydroxyproline residues using YASARA [70]. The GROMACS package v5.0.7 [71] was used to run MD simulations. The AMBER99SB-ILDN [72] force field was used to provide molecular mechanics parameters for our peptides. The peptides were put in a cubic cell (“box”), the border of which was at least 1 nm from the protein, and we solvated it with TIP3P-explicit water molecules. Counterions were added, if necessary, to obtain a neutral system and took the place of water molecules. Energy minimization and the temperature and pressure equilibrations were undertaken as described by Pokotylo et al. [73]. The equilibration time was also set at 100 ps relaxation time. Once our peptide was well-equilibrated at the desired temperature (300 K) and pressure (1 bar), we released the position restraints and ran production MD for data collection. The peptides were subjected to 500 ns simulation with 2 fs time steps. To evaluate the reproducibility, the whole process (minimization, equilibration and production run) was repeated thrice. PyMol [74] and VMD [75] were used for visualisation. Graphs and images were created with GNUplot [76] and PyMol [74]. All MD trajectories were analysed using GROMACS tools [77] along the last 250 ns. Polar contacts maps were determined by calculating the radial distribution function of each nitrogen and oxygen atom in relation to all others and taking its maximum intensity in the range of H-bonds and salt bridges. This made it possible to obtain an overview of all polar interatomic interactions and their occurrence [78].

### 4.6. Seedling Growth Inhibition Assay

Seedlings were germinated on MS (1×) agar (1%) and grown for five days before transferring one seedling into each well of 24-well plates containing 500 μL of MS medium or MS medium supplemented with the indicated elicitor peptide to a final concentration of 1 μM (six replicates per elicitor peptide treatment). Fresh masses were measured and the experiment was repeated thrice.

### 4.7. ROS Assay

ROS production was determined with a luminol-based assay. Three five-week-old seedlings grown on MS plates were incubated in 200 μL double-distilled water (ddH_2_O) overnight in a 1.5 mL centrifuge tube. Then, ddH_2_O was replaced by 200 μL of reaction solution containing 100 μM of luminol and 10 μg/mL of horseradish peroxidase (Sigma-Aldrich, Saint-Louis, USA) supplemented with 1 μM peptide or without supplementation. Luminescence was measured for 60 min at one-second intervals immediately after adding the solution with a FLUOstar OPTIMA plate reader (BMG LABTECH, Ortenberg, Germany). The total values for ROS production were indicated as the means of the relative light units (RLUs).

### 4.8. Gene Expression Analysis

For each of the three biological repetitions, shoot apical meristem (SAM) samples from 15 individual 7 and 11 day old Col-0, *proscoop10-1* and *proscoop10-2* plants grown under long-day conditions were hand-dissected under a binocular magnifier, aiming to remove as much leaf tissue as possible. Total RNAs were extracted using the Nucleospin RNA Plus Kit (Macherey-Nagel, Düren, Germany). cDNAs were synthesized from 1.5 µg of total RNA with oligo(dT) primers using Moloney Murine Leukemia Virus Reverse Transcriptase MMLV-RT according to the manufacturer’s instructions (Promega, Madison, WI, USA). RT-qPCR was carried out in a Chromo4 system (Bio-Rad, Laboratories, CA, USA). Expression profiles of key floral transition genes were calculated using the 2^−∆∆Ct^ method and were corrected as recommended by Vandesompele et al. [79], with three internal reference genes (*ACT2*, *COP1* and *AP4M*) used for the calculation of a normalization factor. The mean expression level of Col-0 at 7 days served as a calibrator. The primers used for the RT-qPCR analysis are specified in Supplementary Table S1.

## Figures and Tables

**Table 1:** Expression profiles of the *PROSCOOP10* genes extracted from public data.

Condition	Phenotype	*PROSCOOP10* Transcription	Reference
10th **vs.** 9th day in SAM wild-type	Wild-type	Down-regulated	[48]
*pif1-5***vs.** wild-type	Early-flowering	Down-regulated	[55,57]
*abi4vtc2***vs.** wild-type	Early-flowering	Down-regulated	[50]
*phyABCDE***vs.** wild-type	Early-flowering	Down-regulated	[51]
Brassinolide treatment in wild-type	Early-flowering	Down-regulated	[45]
MeJA treatment in wild-type	Early-flowering	Down-regulated	[58]
35S::*ARAF1* **vs.** wild-type	Late-flowering	Up-regulated	[59]
*vtc2***vs.** wild-type	Late-flowering	Up-regulated	[52]
*tcp4***vs.** wild-type	Late-flowering	Up-regulated	[53]
*ga1-3***vs.** wild-type	No flowering	Up-regulated	[47]
*arf6-2/arf8-3***vs.** wild-type	Immature flowers	Up-regulated	[54]

## Data Availability

All data supporting the findings of this study are available within the paper and within its Supplementary Materials published online. Biological materials are available from the corresponding authors upon request.

## Supplementary Materials

The following supporting information can be downloaded at: https://www.mdpi.com/article/10.3390/plants11243554/s1. Supplementary Table S1: Primer sets for genotyping; Supplementary Table S2: ^1^H and ^13^C NMR assignment of SCOOP10#2* peptide; 1 mM in 50 mM phosphate buffer (10% D_2_O), pH 6.6, 278 K; Supplementary Table S3: ^1^H and ^13^C NMR assignment of hydroxylated SCOOP10#2 peptide; 0.5 mM in 50 mM phosphate buffer (10% D_2_O), pH 6.6, 278 K; Supplementary Table S4: ^1^H and ^13^C NMR assignment of SCOOP10#2* peptide; 0.5 mM in DMSO, 298 K; Supplementary Table S5: ^1^H and ^13^C NMR assignment of hydroxylated SCOOP10#2 peptide; 0.5 mM in DMSO, 298 K; Supplementary Table S6: ^1^H and ^13^C NMR assignment of hydroxylated SCOOP10#1 peptide; 0.5 mM in 50 mM phosphate buffer (10% D_2_O), pH 6.6, 278 K; Supplementary Table S7: ^1^H and ^13^C NMR assignment of hydroxylated SCOOP10#1 peptide; 0.5 mM in DMSO, 298 K; Supplementary Table S8: Number of bolted inflorescences for Col-0 and *proscoop10* mutants per day after germination and per repetition.
