# Supplementary material for: The PROSCOOP10 Gene Encodes Two Extracellular Hydroxylated Peptides and Impacts Flowering Time in Arabidopsis
Source: Plants (Basel). 2022 Dec 16;11(24):3554. doi: 10.3390/plants11243554 (PMC9784617; doi:10.3390/plants11243554)
Supplement: Supplementary file 1 [file plants-11-03554-s001.zip › plants-2029070-supplementary/Supplementary_Data/Supplementary Data_Figures.pdf]

SUPPLEMENTARY DATA

Supplementary data are available online.

Supplementary Table S1: Primer sets for genotyping

Supplementary Table S2: ^{1}H and ^{13}C NMR assignment of SCOOP10#2* peptide 1 mM in 50 mM phosphate buffer (10% D_2O), pH 6.6, 278K

Supplementary Table S3: ^{1}H and ^{13}C NMR assignment of hydroxylated SCOOP10#2 peptide 0.5 mM in 50 mM phosphate buffer (10% D_2O), pH 6.6, 278 K

Supplementary Table S4: ^{1}H and ^{13}C NMR assignment of SCOOP10#2* peptide 0.5 mM in DMSO, 298K

Supplementary Table S5: ^{1}H and ^{13}C NMR assignment of hydroxylated SCOOP10#2 peptide 0.5 mM in DMSO, 298K

Supplementary Table S6: ^{1}H and ^{13}C NMR assignment of hydroxylated SCOOP10#1 peptide 0.5 mM in 50 mM phosphate buffer (10% D_2O), pH 6.6, 278K

Supplementary Table S7: ^{1}H and ^{13}C NMR assignment of hydroxylated SCOOP10#1 peptide 0.5 mM in DMSO, 298K

Supplementary Table S8: Number of bolted inflorescences for Col-0 and *proscoop10* mutants per day after germination and per repetition

Supplementary Figure S1: Genotyping of *proscoop10* mutant lines

Supplementary Figure S2: MS data related to SCOOP10#1 and SCOOP10#2 originating from PROSCOOP10 identified in rosette leaves apoplastic fluids

Supplementary Figure S3: NMR assignment and cis/trans isomerization of SCOOP10#2 in DMSO

Supplementary Figure S4: MD simulations of SCOOP10#2 mutants

Supplementary Figure S5: Representative structures found in MD simulations for SCOOP10#2 and its mutants

Supplementary Figure S6: Structural behaviour of SCOOP10#1 in solution as monitored by NMR

Supplementary Figure S7: Secondary structures and intramolecular interactions found in MD simulations of SCOOP10#1

Supplementary Figure S1: Genotyping of *proscoop10* mutant lines.

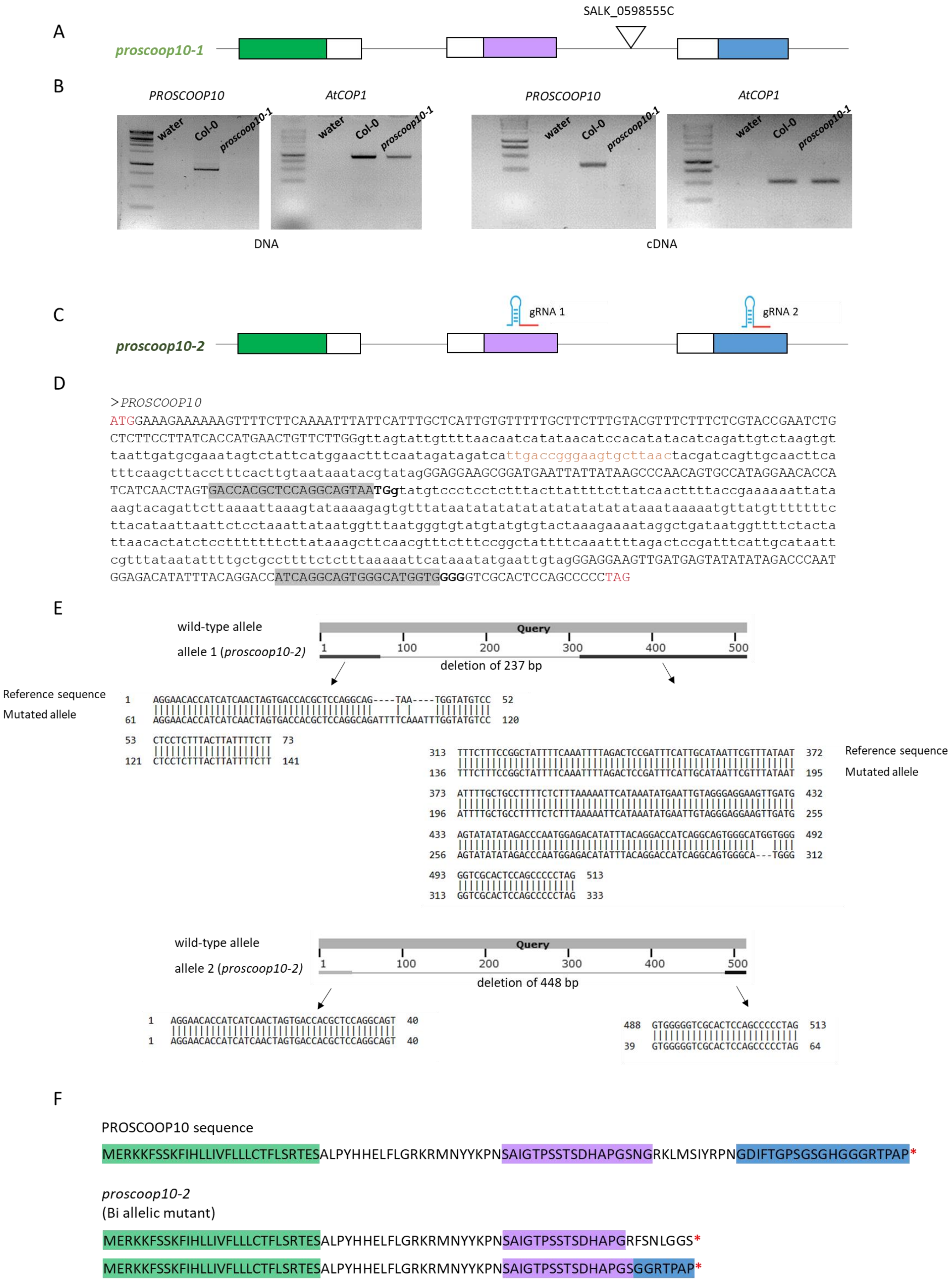

Supplementary Figure S1: Genotyping of *proscoop10* mutant lines

(**A**) Position of the T-DNA insertion in the *proscoop10-1* mutant line. (**B**) On the left, check of the T-DNA insertion by PCR on *PROSCOOP10* and comparison with PCR on *AtCOP1*, used as a PCR positive control, in DNA of Col-0 and *proscoop10-1* mutant line. On the right, check of *PROSCOOP10* expression impairment in *proscoop10-1* mutant line compared to Col-0 wild-type, with *AtCOP1* used at RT-PCR positive control. (**C**) Position of the two RNA guides targeting SCOOP10#1 and SCOOP10#2 regions in the *proscoop10-2* mutant line. (**D**) Reference sequence of PROSCOOP10. In red, the start and stop codons ; in orange, the primer used for PCR amplification and sequencing after cloning; highlighted in grey, the guides RNAg1 and RNAg2 and in bold, the PAM1 and PAM2. (**E**) Sequencing revealed two different alleles in the therefore bi allelic *proscoop10-2* mutant. Alignments with the reference sequence shown for the first mutated allelic version a deletion of 237 bp between the two guides, in addition to nucleotide modifications in guides RNAg1 and RNAg2. This led to a cut into SCOOP10#1 sequence and a frameshift generating a stop codon and eliminating the SCOOP10#2 sequence. For the second mutated allelic version, an important deletion of 448 bp has been generated between the two guides, leading to a break in the two sequences corresponding to SCOOP10#1 and SCOOP10#2 and a fusion of the remaining sequences. (**F**) Amino acid sequences of PROSCOOP10 in *proscoop10-2* bi allelic mutant after CRISPR/Cas9 edition. For (**A**) and (**C**) exons (CDS) and introns are represented by boxes and lines respectively. For (**A**), (**C**) and (**F**) purple: SCOOP10#1 region; blue: SCOOP10#2 region; green: signal peptide; red star: stop codon.

Supplementary Figure S2. MS data related to SCOOP10#1 and SCOOP10#2 originating from PROSCOOP10 identified in rosette leaves apoplastic fluids.

A. MS/MS data for SAIGTOSSTSDHAOGSNG

B. MS/MS data for GDIFTGPSGSGHGGGR

C. MS/MS data for GDIFTGOSGSGHGGGR

D. MS/MS data for GDIFTGPSGSGHGGGRTO

E. MS/MS data for GDIFTGPSGSGHGGGRTOA

F. MS/MS data for GDIFTGOSGSGHGGGRTO

G. MS/MS data for GDIFTGOSGSGHGGGRTOA

H. MS/MS data for GDIFTGPSGSGHGGGRTPAO

I. MS/MS data for GDIFTGOSGSGHGGGRTPAO

(**A-I**) For each peptide, a representative MS/MS spectrum is shown. The positions of the hydroxyproline (O)/proline (P) residues are highlighted using the fragmentation data from the C-terminus of each peptide (y ions). The information describing the experimental procedure is available in Material and Methods.

A. MS/MS data for SAIGTOSSTSDHAOGSNG

y ions

Sample information

#	log(e)	log(I)	m+h	delta	ζ	sequence
2706	-7.0	6.20	1674.7206	-0.0005	2/2	ykpn[51] SAIGTPSSTSDHAPGSNG[68]rklm

mods: P56+Oxidation, P64+Oxidation

20211021_stresspep_test_11_211025225206.mzXML scan 2706 (charge 2)

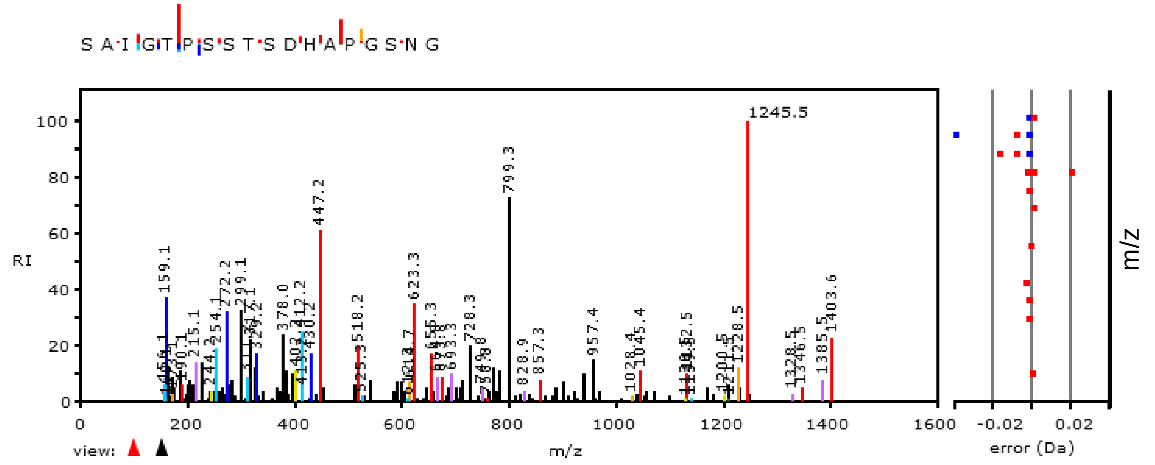

matched/total: # ions: 28% intensity: 47% μ: -0.00, σ: 0.0094 Da

bond	+1y	+1y-17	+1y-18	+1b	+1b-17	+1b-18
S1	1587.688	1570.662	1569.678	88.039	71.013	70.029
A2	+2 1516.651	1499.625	1498.641	159.077	142.050	141.066
I3	1403.567	1386.541	1385.557	272.161	255.134	254.150
G4	+1,2 1346.546	1329.519	1328.535	329.182	312.156	311.171
T5	+1,2 1245.498	1228.472	1227.488	430.230	413.203	412.219
P6	1132.450	1115.424	1114.440	+2 543.277	526.251	525.267
S7	1045.418	1028.392	1027.408	630.309	613.283	612.299
S8	958.386	941.360	940.376	717.341	700.315	699.331
T9	857.339	840.312	839.328	818.389	801.363	800.379
S10	770.307	753.280	752.296	905.421	888.395	887.411
D11	655.280	638.253	637.269	1020.448	1003.422	1002.438
H12	518.221	501.194	500.210	1157.507	1140.480	1139.496
A13	447.184	430.157	429.173	1228.544	1211.518	1210.534
P14	334.136	317.109	316.125	1341.592	1324.565	1323.581
G15	277.115	260.088	259.104	1398.613	1381.587	1380.603
S16	190.083	173.056	172.072	1485.645	1468.619	1467.635
N17	76.040	59.013	58.029	1599.688	1582.662	1581.678

Hyp (O): 113 Da (T5–P6)

Hyp (O): 113 Da (A13–P14)

B. MS/MS data for GDIFTG<u>P</u>SGSGHGGGR

y ions ←

Sample information

#	log(e)	log(I)	m+h	delta	ζ	sequence
6477	-5.9	5.37	1458.6725	-0.0009	3/3	yrpn[79] GDIFTGPSGSGHGGGR[94]tpap]

20220322_stresspep_apo_P2_dig.mzXML scan 6477 (charge 3)

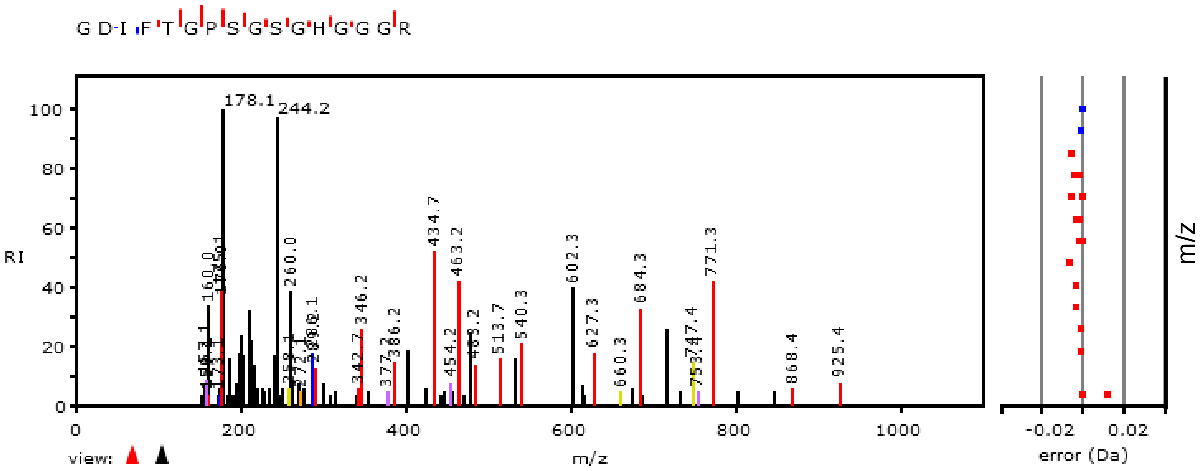

matched/total: # ions: 31% intensity: 34% μ: -0.00, σ: 0.0020 Da

Pro (P): 97 Da { (G6, P7)

bond	+1y	+1y-17	+1y-18	+1b	+1b-17	+1b-18	+2y	+2y-17	+2y-18	+2b	+2b-17	+2b-18
G1	1401.651	1384.624	1383.640	58.029	41.002	40.018	701.329	692.816	692.324	29.518	21.005	20.513
D2	1286.624	1269.597	1268.613	173.056	156.029	155.045	643.816	635.302	634.810	87.032	78.518	78.026
I3	1173.540	1156.513	1155.529	286.140	269.113	268.129	587.274	578.760	578.268	143.574	135.060	134.568
F4	1026.471	1009.445	1008.461	433.208	416.182	415.198	513.739	505.226	504.734	217.108	208.595	208.102
T5	925.424	908.397	907.413	534.256	517.229	516.245	463.216	454.702	454.210	267.632	259.118	258.626
G6	868.402	851.376	850.392	591.277	574.251	573.267	434.705	426.192	425.700	296.142	287.629	287.137
P7	771.350	754.323	753.339	688.330	671.304	670.320	386.178	377.665	377.173	344.669	336.155	335.663
S8	684.318	667.291	666.307	775.362	758.336	757.352	342.662	334.149	333.657	388.185	379.671	379.179
G9	627.296	610.270	609.285	832.384	815.357	814.373	314.152	305.638	305.146	416.695	408.182	407.690
S10	540.264	523.237	522.253	919.416	902.389	901.405	270.636	262.122	261.630	460.211	451.698	451.206
G11	483.243	466.216	465.232	976.437	959.411	958.427	242.125	233.612	233.120	488.722	480.209	479.717
H12	346.184	329.157	328.173	1113.496	1096.470	1095.486	173.595	165.082	164.590	557.252	548.738	548.246
G13	289.162	272.136	271.152	1170.518	1153.491	1152.507	145.085	136.571	136.079	585.762	577.249	576.757
G14	232.141	215.114	214.130	1227.539	1210.512	1209.528	116.574	108.061	107.569	614.273	605.760	605.268
G15	175.119	158.093	157.109	1284.560	1267.534	1266.550	88.063	79.550	79.058	642.784	634.271	633.779

C. MS/MS data for GDIFTGOSGSGHGGGR

y ions

Sample information

#	log(e)	log(I)	m+h	delta	ζ	sequence
4660	-4.9	5.32	1474.6674	0.0030	3/3	yrpn[79] GDIFTGPSGSGHGGGR[94]tpap]

mods: P85+Oxidation

20220322_stresspep_apo_P2_dig.mzXML scan 4660 (charge 3)

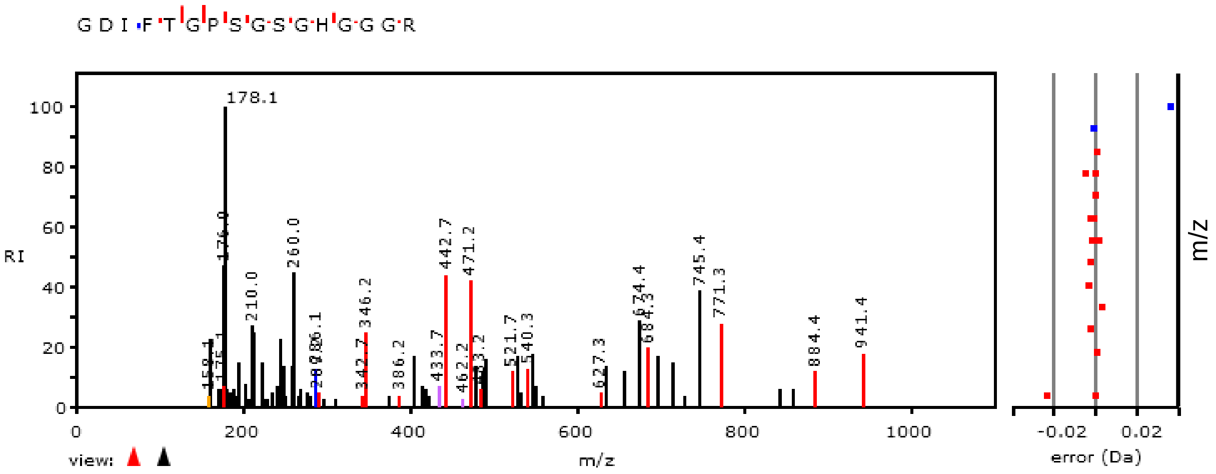

matched/total: # ions: 22% intensity: 26% µ: 0.00, σ: 0.0084 Da

Hyp (O): 113 Da (G6–P7)

bond	+1y	+1y-17	+1y-18	+1b	+1b-17	+1b-18	+2y	+2y-17	+2y-18	+2b	+2b-17	+2b-18
G1	1417.646	1400.619	1399.635	58.029	41.002	40.018	709.327	700.813	700.321	29.518	21.005	20.513
D2	1302.619	1285.592	1284.608	173.056	156.029	155.045	651.813	643.300	642.808	87.032	78.518	78.026
I3	1189.535	1172.508	1171.524	286.140	269.113	268.129	595.271	586.758	586.266	143.574	135.060	134.568
F4	1042.466	1025.440	1024.456	433.208	416.182	415.198	521.737	513.224	512.732	217.108	208.595	208.102
T5	941.419	924.392	923.408	534.256	517.229	516.245	471.213	462.700	462.208	267.632	259.118	258.626
G6	884.397	867.371	866.387	591.277	574.251	573.267	442.702	434.189	433.697	296.142	287.629	287.137
P7	771.350	754.323	753.339	704.325	687.299	686.315	386.178	377.665	377.173	352.666	344.153	343.661
S8	684.318	667.291	666.307	791.357	774.331	773.347	342.662	334.149	333.657	396.182	387.669	387.177
G9	627.296	610.270	609.285	848.379	831.352	830.368	314.152	305.638	305.146	424.693	416.180	415.688
S10	540.264	523.237	522.253	935.411	918.384	917.400	270.636	262.122	261.630	468.209	459.696	459.204
G11	483.243	466.216	465.232	992.432	975.406	974.422	242.125	233.612	233.120	496.720	488.206	487.714
H12	346.184	329.157	328.173	1129.491	1112.464	1111.480	173.595	165.082	164.590	565.249	556.736	556.244
G13	289.162	272.136	271.152	1186.512	1169.486	1168.502	145.085	136.571	136.079	593.760	585.247	584.755
G14	232.141	215.114	214.130	1243.534	1226.507	1225.523	116.574	108.061	107.569	622.271	613.757	613.265
G15	175.119	158.093	157.109	1300.555	1283.529	1282.545	88.063	79.550	79.058	650.781	642.268	641.776

D. MS/MS data for GDIFTGPSGSGHGGGRTO

y ions ←

Sample information

#	log(e)	log(I)	m+h	delta	ζ	sequence
4969	-7.1	5.59	1672.7678	-0.0010	3/3	yrpn[79] GDIFTGPSGSGHGGGRTP[96]ap]

mods: P96+Oxidation

20200221_RD_jamet_9_mono.mzXML scan 4969 (charge 3)

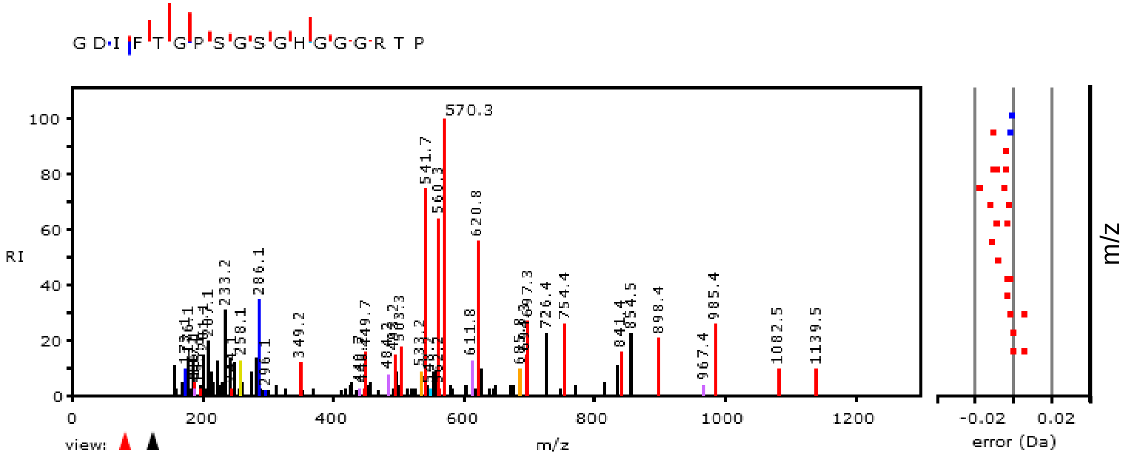

matched/total: # ions: 28% intensity: 56% μ: -0.00, σ: 0.0059 Da

bond	+1 y	+1 y-17	+1 y-18	+1 b	+1 b-17	+1 b-18	+2 y	+2 y-17	+2 y-18	+2 b	+2 b-17	+2 b-18
G1	1615.746	1598.720	1597.736	58.029	41.002	40.018	808.377	799.863	799.371	29.518	21.005	20.513
D2	1500.719	1483.693	1482.709	173.056	156.029	155.045	750.863	742.350	741.858	87.032	78.518	78.026
I3	1387.635	1370.609	1369.625	286.140	269.113	268.129	694.321	685.808	685.316	143.574	135.060	134.568
F4	1240.567	1223.540	1222.556	433.208	416.182	415.198	620.787	612.274	611.782	217.108	208.595	208.102
T5	1139.519	1122.493	1121.509	534.256	517.229	516.245	570.263	561.750	561.258	267.632	259.118	258.626
G6	1082.498	1065.471	1064.487	591.277	574.251	573.267	541.752	533.239	532.747	296.142	287.629	287.137
P7	985.445	968.418	967.434	688.330	671.304	670.320	493.226	484.713	484.221	344.669	336.155	335.663
S8	898.413	881.386	880.402	775.362	758.336	757.352	449.710	441.197	440.705	388.185	379.671	379.179
G9	841.391	824.365	823.381	832.384	815.357	814.373	421.199	412.686	412.194	416.695	408.182	407.690
S10	754.359	737.333	736.349	919.416	902.389	901.405	377.683	369.170	368.678	460.211	451.698	451.206
G11	697.338	680.311	679.327	976.437	959.411	958.427	349.173	340.659	340.167	488.722	480.209	479.717
H12	560.279	543.252	542.268	1113.496	1096.470	1095.486	280.643	272.130	271.638	557.252	548.738	548.246
G13	503.258	486.231	485.247	1170.518	1153.491	1152.507	252.132	243.619	243.127	585.762	577.249	576.757
G14	446.236	429.210	428.226	1227.539	1210.512	1209.528	223.622	215.108	214.616	614.273	605.760	605.268
G15	389.215	372.188	371.204	1284.560	1267.534	1266.550	195.111	186.598	186.106	642.784	634.271	633.779
R16	233.114	216.087	215.103	1440.662	1423.635	1422.651	117.060	108.547	108.055	720.834	712.321	711.829
T17	132.066	115.039	114.055	1541.709	1524.683	1523.699	66.537	58.023	57.531	771.358	762.845	762.353

Pro (P): 97 Da { G6 / P7

Hyp (O): 132 Da { T17

E. MS/MS data for GDIFTGPSGSGHGGGRTOA

Sample information

#	log(e)	log(I)	m+h	delta	ζ	sequence
4937	-9.2	6.08	1743.8049	-0.0028	2/3	yrpn[79] GDIFTGPSGSGHGGGRTPA[97]p]

y ions

mods: P96+Oxidation

20211021_stresspep_test_8_211026040406.mzXML scan 4937 (charge 2)

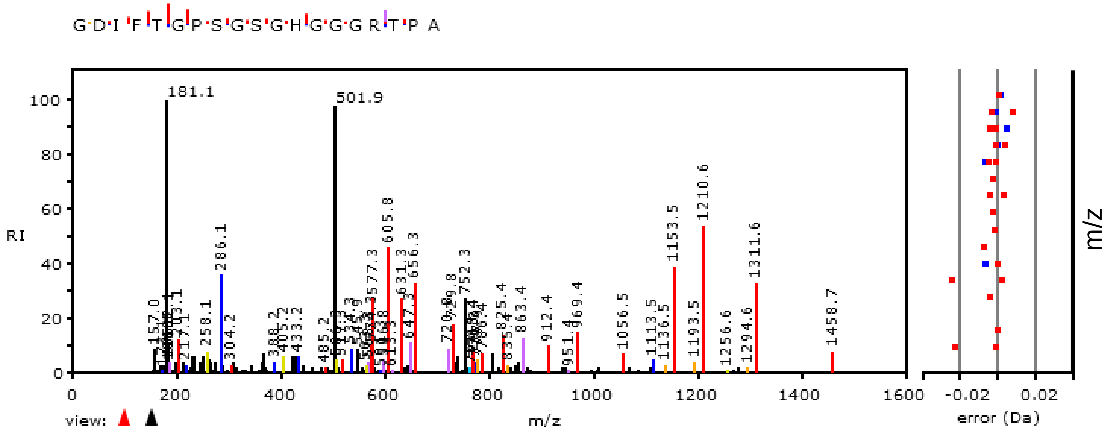

matched/total: # ions: 33% intensity: 52% µ: -0.00, σ: 0.0054 Da

bond	+1 y	+1 y-17	+1 y-18	+1 b	+1 b-17	+1 b-18
G_1	1686.783	1669.757	1668.773	58.029	41.002	40.018
D_2	+2 1571.756	1554.730	1553.746	173.056	156.029	155.045
I_3	+1,2 1458.672	1441.646	1440.662	286.140	269.113	268.129
F_4	+1,2 1311.604	1294.577	1293.593	+1,2 433.208	416.182	415.198
T_5	+1,2 1210.556	1193.530	1192.546	534.256	517.229	516.245
G_6	+1,2 1153.535	1136.508	1135.524	591.277	574.251	573.267
P_7	1056.482	1039.455	1038.471	688.330	671.304	670.320
S_8	+1,2 969.450	952.423	951.439	+2 775.362	758.336	757.352
G_9	912.429	895.402	894.418	832.384	815.357	814.373
S_{10}	825.396	808.370	807.386	919.416	902.389	901.405
G_{11}	768.375	751.348	750.364	976.437	959.411	958.427
H_{12}	631.316	614.290	613.306	1113.496	1096.470	1095.486
G_{13}	574.295	557.268	556.284	1170.518	1153.491	1152.507
G_{14}	517.273	500.247	499.263	1227.539	1210.512	1209.528
G_{15}	460.252	443.225	442.241	1284.560	1267.534	1266.550
R_{16}	304.151	287.124	286.140	+2 1440.662	1423.635	1422.651
T_{17}	203.103	186.076	185.092	+2 1541.709	1524.683	1523.699
P_{18}	90.055	73.029	72.045	1654.757	1637.730	1636.746

Pro (P): 97 Da { (G_6, P_7)

Hyp (O): 113 Da { (T_{17}, P_{18})

F. MS/MS data for GDIFTGOSGSGHGGGRTO

y ions

Sample information

#	log(e)	log(I)	m+h	delta	ζ	sequence
3574	-8.1	6.19	1688.7627	0.0029	3/3	yrpn[79] GDIFTGPSGSGHGGGRTP[96]ap]

mods: P85+Oxidation, P96+Oxidation

20200221_RD_jamet_12_mono.mzXML scan 3574 (charge 3)

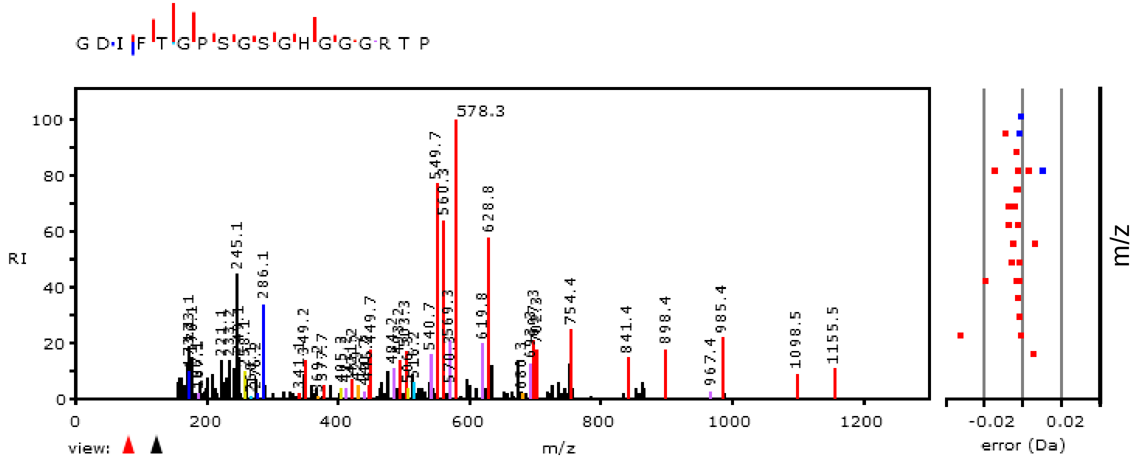

matched/total: # ions: 28% intensity: 55% μ: -0.00, σ: 0.0078 Da

	bond	+1y	+1y-17	+1y-18	+1b	+1b-17	+1b-18	+2y	+2y-17	+2y-18	+2b	+2b-17	+2b-18
	G1	1631.741	1614.715	1613.731	58.029	41.002	40.018	816.374	807.861	807.369	29.518	21.005	20.513
	D2	1516.714	1499.688	1498.704	173.056	156.029	155.045	758.861	750.347	749.855	87.032	78.518	78.026
	I3	1403.630	1386.604	1385.620	286.140	269.113	268.129	702.319	693.805	693.313	143.574	135.060	134.568
	F4	1256.562	1239.535	1238.551	433.208	416.182	415.198	628.784	620.271	619.779	217.108	208.595	208.102
	T5	1155.514	1138.487	1137.503	534.256	517.229	516.245	578.261	569.747	569.255	267.632	259.118	258.626
Hyp (O): 113 Da	G6	1098.493	1081.466	1080.482	591.277	574.251	573.267	549.750	541.237	540.745	296.142	287.629	287.137
	P7	985.445	968.418	967.434	704.325	687.299	686.315	493.226	484.713	484.221	352.666	344.153	343.661
	S8	898.413	881.386	880.402	791.357	774.331	773.347	449.710	441.197	440.705	396.182	387.669	387.177
	G9	841.391	824.365	823.381	848.379	831.352	830.368	421.199	412.686	412.194	424.693	416.180	415.688
	S10	754.359	737.333	736.349	935.411	918.384	917.400	377.683	369.170	368.678	468.209	459.696	459.204
	G11	697.338	680.311	679.327	992.432	975.406	974.422	349.173	340.659	340.167	496.720	488.206	487.714
	H12	560.279	543.252	542.268	1129.491	1112.464	1111.480	280.643	272.130	271.638	565.249	556.736	556.244
	G13	503.258	486.231	485.247	1186.512	1169.486	1168.502	252.132	243.619	243.127	593.760	585.247	584.755
	G14	446.236	429.210	428.226	1243.534	1226.507	1225.523	223.622	215.108	214.616	622.271	613.757	613.265
	G15	389.215	372.188	371.204	1300.555	1283.529	1282.545	195.111	186.598	186.106	650.781	642.268	641.776
	R16	233.114	216.087	215.103	1456.656	1439.630	1438.646	117.060	108.547	108.055	728.832	720.319	719.827
Hyp (O): 132 Da	T17	132.066	115.039	114.055	1557.704	1540.678	1539.694	66.537	58.023	57.531	779.356	770.842	770.350

G. MS/MS data for GDIFTGOSGSGHGGGRTOA

y ions ←

Sample information

#	log(e)	log(I)	m+h	delta	ζ	sequence
3566	-7.1	6.55	1759.7998	-0.0029	3/3	yrpn[79] GDIFTGPSGSGHGGGRTPA[97]p]

mods: P85+Oxidation, P96+Oxidation

20200221_RD_jamet_12_mono.mzXML scan 3566 (charge 3)

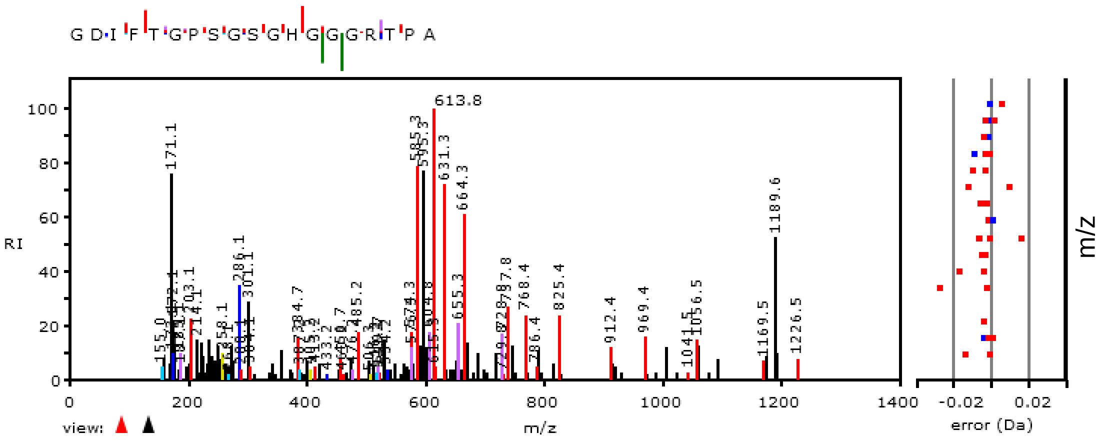

matched/total: # ions: 30% intensity: 45% μ: -0.00, σ: 0.0049 Da

	bond	+1y	+1y-17	+1y-18	+1b	+1b-17	+1b-18	+2y	+2y-17	+2y-18	+2b	+2b-17	+2b-18
	G1	1702.778	1685.752	1684.768	58.029	41.002	40.018	851.893	843.379	842.887	29.518	21.005	20.513
	D2	1587.751	1570.725	1569.741	173.056	156.029	155.045	794.379	785.866	785.374	87.032	78.518	78.026
	I3	1474.667	1457.641	1456.657	286.140	269.113	268.129	737.837	729.324	728.832	143.574	135.060	134.568
	F4	1327.599	1310.572	1309.588	433.208	416.182	415.198	664.303	655.790	655.298	217.108	208.595	208.102
	T5	1226.551	1209.525	1208.541	534.256	517.229	516.245	613.779	605.266	604.774	267.632	259.118	258.626
Hyp (O): 113 Da	G6	1169.530	1152.503	1151.519	591.277	574.251	573.267	585.268	576.755	576.263	296.142	287.629	287.137
	P7	1056.482	1039.455	1038.471	704.325	687.299	686.315	528.745	520.231	519.739	352.666	344.153	343.661
	S8	969.450	952.423	951.439	791.357	774.331	773.347	485.229	476.715	476.223	396.182	387.669	387.177
	G9	912.429	895.402	894.418	848.379	831.352	830.368	456.718	448.205	447.713	424.693	416.180	415.688
	S10	825.396	808.370	807.386	935.411	918.384	917.400	413.202	404.689	404.197	468.209	459.696	459.204
	G11	768.375	751.348	750.364	992.432	975.406	974.422	384.691	376.178	375.686	496.720	488.206	487.714
	H12	631.316	614.290	613.306	1129.491	1112.464	1111.480	316.162	307.648	307.156	565.249	556.736	556.244
	G13	574.295	557.268	556.284	1186.512	1169.486	1168.502	287.651	279.138	278.646	593.760	585.247	584.755
	G14	517.273	500.247	499.263	1243.534	1226.507	1225.523	259.140	250.627	250.135	622.271	613.757	613.265
	G15	460.252	443.225	442.241	1300.555	1283.529	1282.545	230.630	222.116	221.624	650.781	642.268	641.776
	R16	304.151	287.124	286.140	1456.656	1439.630	1438.646	152.579	144.066	143.574	728.832	720.319	719.827
Hyp (O): 113 Da	T17	203.103	186.076	185.092	1557.704	1540.678	1539.694	102.055	93.542	93.050	779.356	770.842	770.350
	P18	90.055	73.029	72.045	1670.752	1653.725	1652.741	45.531	37.018	36.526	835.880	827.366	826.874

H. MS/MS data for GDIFTGPSGSGHGGGRTPAO

y ions

Sample information

#	log(e)	log(I)	m+h	delta	ζ	sequence
5057	-2.7	5.27	1840.8577	0.0044	3/3	yrpn[79] GDIFTGPSGSGHGGGRTPAP[98]]

mods: P98+Oxidation

20200221_RD_jamet_09.mzXML scan 5057 (charge 3)

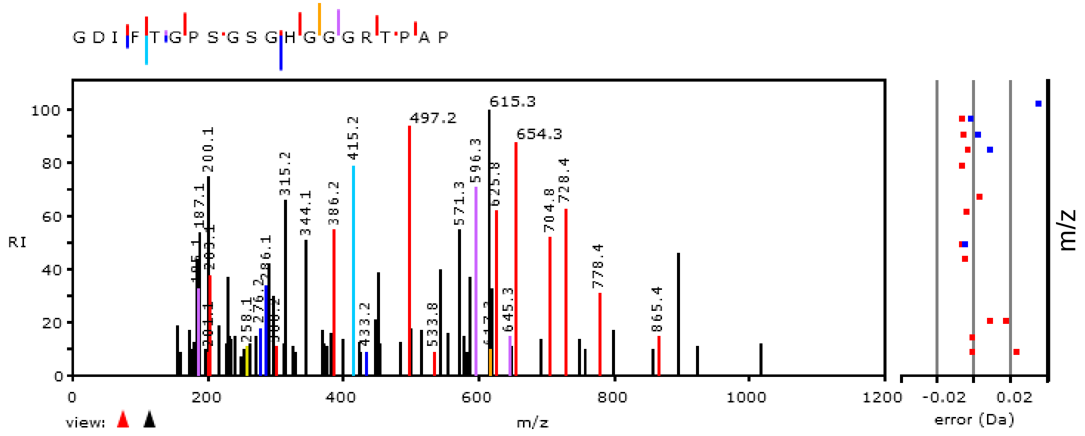

matched/total: # ions: 26% intensity: 37% μ: 0.00, σ: 0.0098 Da

bond	+1y	+1y-17	+1y-18	+1b	+1b-17	+1b-18	+2y	+2y-17	+2y-18	+2b	+2b-17	+2b-18
G1	1783.836	1766.810	1765.826	58.029	41.002	40.018	892.422	883.908	883.416	29.518	21.005	20.513
D2	1668.809	1651.783	1650.799	173.056	156.029	155.045	834.908	826.395	825.903	87.032	78.518	78.026
I3	1555.725	1538.699	1537.715	286.140	269.113	268.129	778.366	769.853	769.361	143.574	135.060	134.568
F4	1408.657	1391.630	1390.646	433.208	416.182	415.198	704.832	696.319	695.827	217.108	208.595	208.102
T5	1307.609	1290.582	1289.598	534.256	517.229	516.245	654.308	645.795	645.303	267.632	259.118	258.626
G6	1250.588	1233.561	1232.577	591.277	574.251	573.267	625.797	617.284	616.792	296.142	287.629	287.137
P7	1153.535	1136.508	1135.524	688.330	671.304	670.320	577.271	568.758	568.266	344.669	336.155	335.663
S8	1066.503	1049.476	1048.492	775.362	758.336	757.352	533.755	525.242	524.750	388.185	379.671	379.179
G9	1009.481	992.455	991.471	832.384	815.357	814.373	505.244	496.731	496.239	416.695	408.182	407.690
S10	922.449	905.423	904.439	919.416	902.389	901.405	461.728	453.215	452.723	460.211	451.698	451.206
G11	865.428	848.401	847.417	976.437	959.411	958.427	433.218	424.704	424.212	488.722	480.209	479.717
H12	728.369	711.342	710.358	1113.496	1096.470	1095.486	364.688	356.175	355.683	557.252	548.738	548.246
G13	671.347	654.321	653.337	1170.518	1153.491	1152.507	336.177	327.664	327.172	585.762	577.249	576.757
G14	614.326	597.299	596.315	1227.539	1210.512	1209.528	307.667	299.153	298.661	614.273	605.760	605.268
G15	557.304	540.278	539.294	1284.560	1267.534	1266.550	279.156	270.643	270.151	642.784	634.271	633.779
R16	401.203	384.177	383.193	1440.662	1423.635	1422.651	201.105	192.592	192.100	720.834	712.321	711.829
T17	300.156	283.129	282.145	1541.709	1524.683	1523.699	150.581	142.068	141.576	771.358	762.845	762.353
P18	203.103	186.076	185.092	1638.762	1621.735	1620.751	102.055	93.542	93.050	819.885	811.371	810.879
A19	132.066	115.039	114.055	1709.799	1692.773	1691.789	66.537	58.023	57.531	855.403	846.890	846.398

Pro (P): 97 Da (G6–P7)

Pro (P): 97 Da (T17–P18)

Hyp (O): 132 Da (A19)

I. MS/MS data for GDIFTGOSGSGHGGGRTPAO

Sample information

y ions

#	log(e)	log(I)	m+h	delta	ζ	sequence
6361	-3.2	5.07	1856.8526	0.0015	3/3	yrpn[79] GDIFTGPSGSGHGGGRTPAP[98]]

mods: P85+Oxidation, P98+Oxidation

20220322_stresspep_apo_P2_nodig_220325161114.mzXML scan 6361 (charge 3)

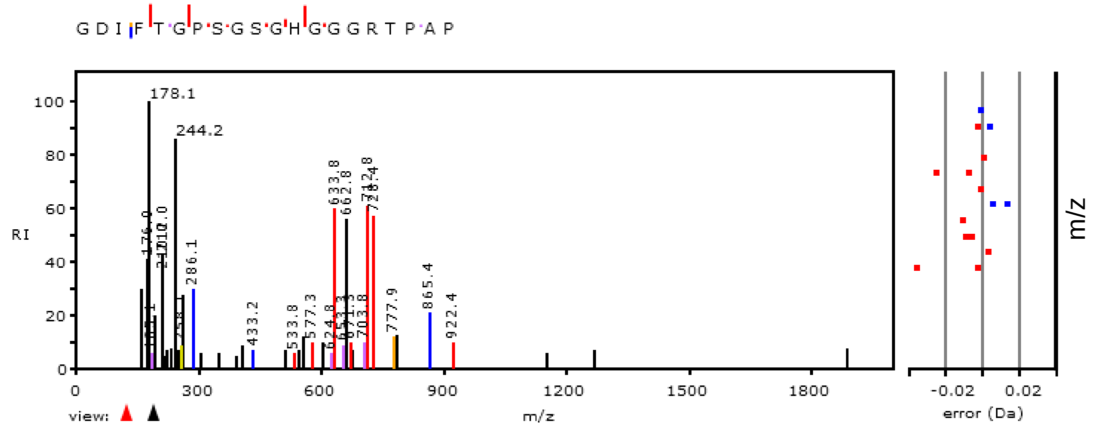

matched/total: # ions: 35% intensity: 35% μ: -0.00, σ: 0.0081 Da

bond	+1 y	+1 y-17	+1 y-18	+1 b	+1 b-17	+1 b-18	+2 y	+2 y-17	+2 y-18	+2 b	+2 b-17	+2 b-18
G_1	1799.831	1782.804	1781.820	58.029	41.002	40.018	900.419	891.906	891.414	29.518	21.005	20.513
D_2	1684.804	1667.778	1666.794	173.056	156.029	155.045	842.906	834.392	833.900	87.032	78.518	78.026
I_3	1571.720	1554.693	1553.709	286.140	269.113	268.129	786.364	777.850	777.358	143.574	135.060	134.568
F_4	1424.652	1407.625	1406.641	433.208	416.182	415.198	712.829	704.316	703.824	217.108	208.595	208.102
T_5	1323.604	1306.577	1305.593	534.256	517.229	516.245	662.306	653.792	653.300	267.632	259.118	258.626
G_6	1266.582	1249.556	1248.572	591.277	574.251	573.267	633.795	625.282	624.790	296.142	287.629	287.137
P_7	1153.535	1136.508	1135.524	704.325	687.299	686.315	577.271	568.758	568.266	352.666	344.153	343.661
S_8	1066.503	1049.476	1048.492	791.357	774.331	773.347	533.755	525.242	524.750	396.182	387.669	387.177
G_9	1009.481	992.455	991.471	848.379	831.352	830.368	505.244	496.731	496.239	424.693	416.180	415.688
S_{10}	922.449	905.423	904.439	935.411	918.384	917.400	461.728	453.215	452.723	468.209	459.696	459.204
G_{11}	865.428	848.401	847.417	992.432	975.406	974.422	433.218	424.704	424.212	496.720	488.206	487.714
H_{12}	728.369	711.342	710.358	1129.491	1112.464	1111.480	364.688	356.175	355.683	565.249	556.736	556.244
G_{13}	671.347	654.321	653.337	1186.512	1169.486	1168.502	336.177	327.664	327.172	593.760	585.247	584.755
G_{14}	614.326	597.299	596.315	1243.534	1226.507	1225.523	307.667	299.153	298.661	622.271	613.757	613.265
G_{15}	557.304	540.278	539.294	1300.555	1283.529	1282.545	279.156	270.643	270.151	650.781	642.268	641.776
R_{16}	401.203	384.177	383.193	1456.656	1439.630	1438.646	201.105	192.592	192.100	728.832	720.319	719.827
T_{17}	300.156	283.129	282.145	1557.704	1540.678	1539.694	150.581	142.068	141.576	779.356	770.842	770.350
P_{18}	203.103	186.076	185.092	1654.757	1637.730	1636.746	102.055	93.542	93.050	827.882	819.369	818.877
A_{19}	132.066	115.039	114.055	1725.794	1708.767	1707.783	66.537	58.023	57.531	863.401	854.887	854.395

Hyp (O): 113 Da { G_6, P_7 }

Pro (P): 97 Da { T_{17}, P_{18} }

Hyp (O): 132 Da { A_{19} }

Supplementary Figure S3: NMR assignment and cis/trans isomerization of SCOOP10#2 in DMSO.

A

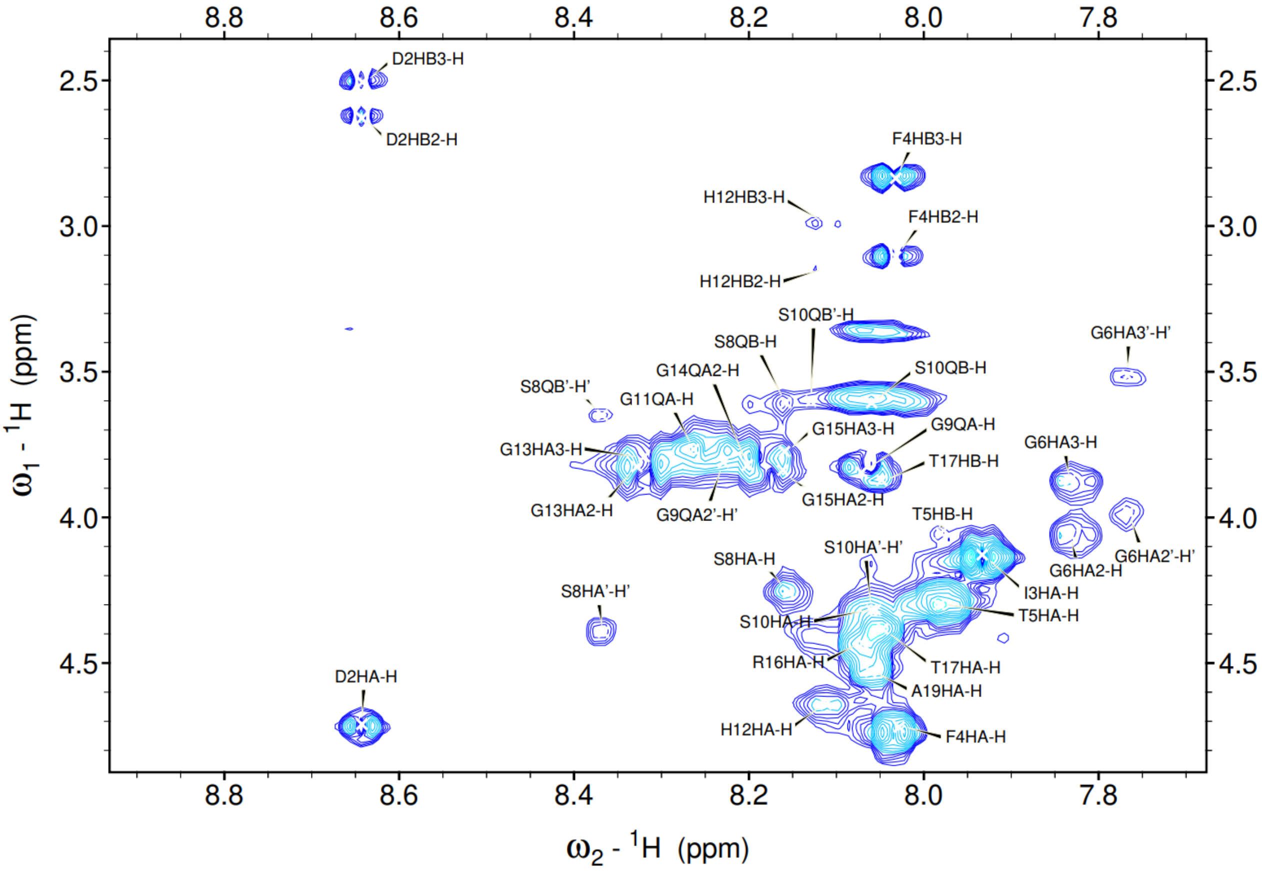

B

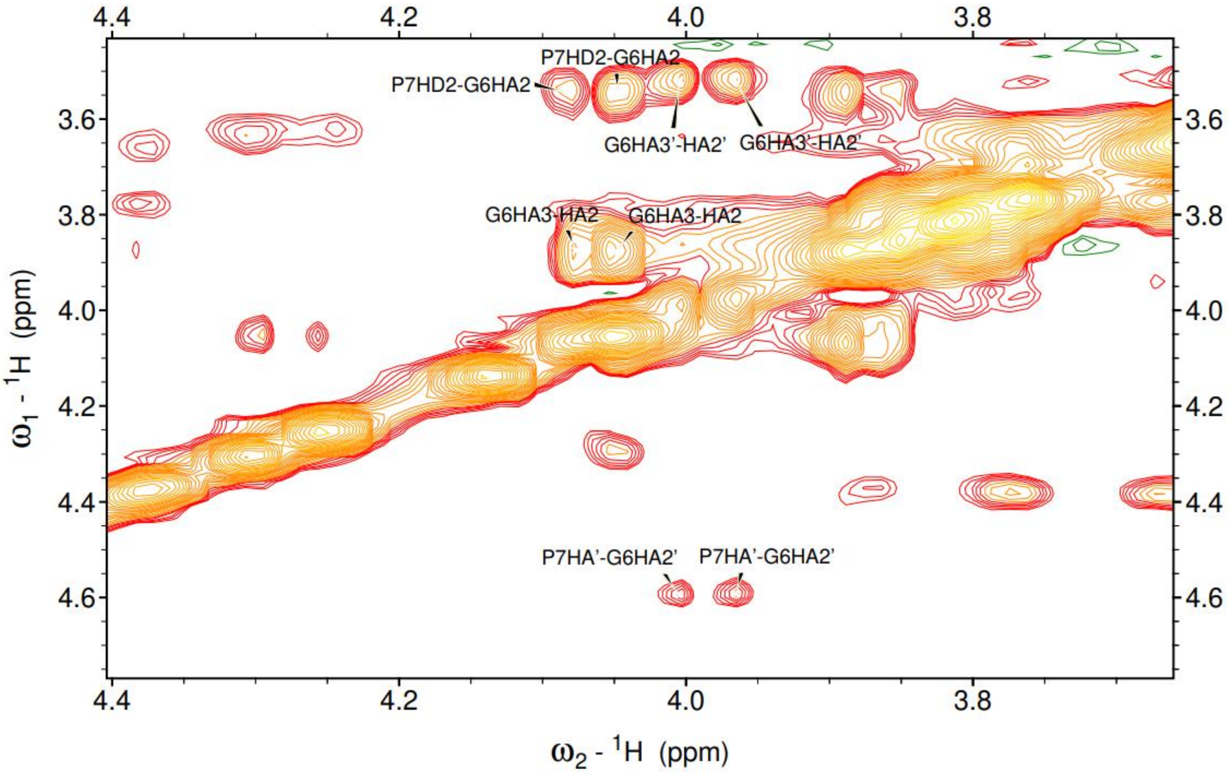

Supplementary Figure S3: NMR assignment and cis/trans isomerization of SCOOP10#2 in DMSO.

(**A**) NMR assignment of SCOOP10#2 reported on 1H,1H-TOCSY spectrum (HN/Hα region). (**B**) 1H,1H-NOESY cross-peaks between Hα protons of G6 and Hα or Hα protons of hydroxyproline in position 7 indicating the presence of both cis and trans conformation of the G6-P7 peptide bond, respectively.

Supplementary Figure S4: MD simulations of SCOOP10#2 mutants.

A

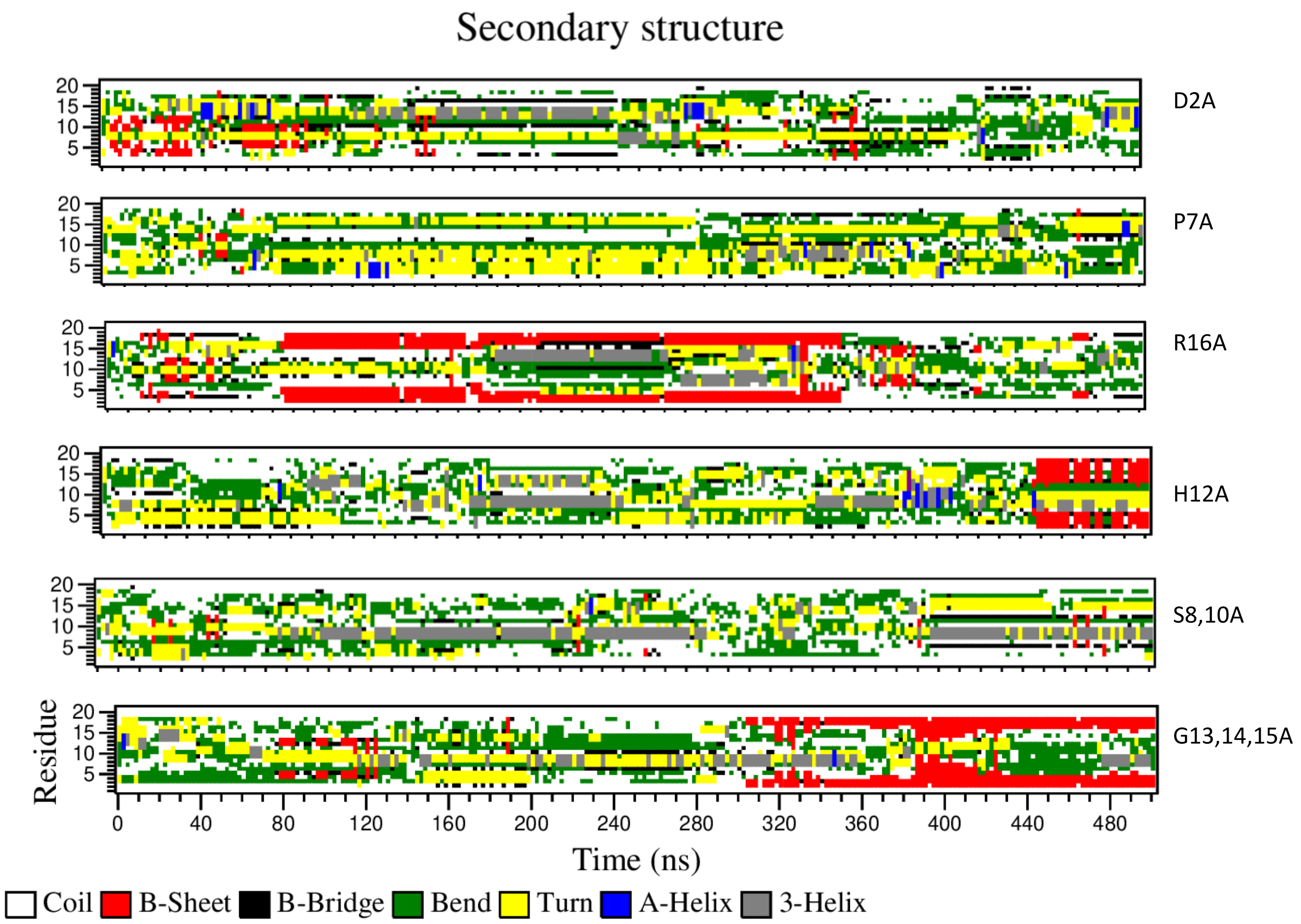

B

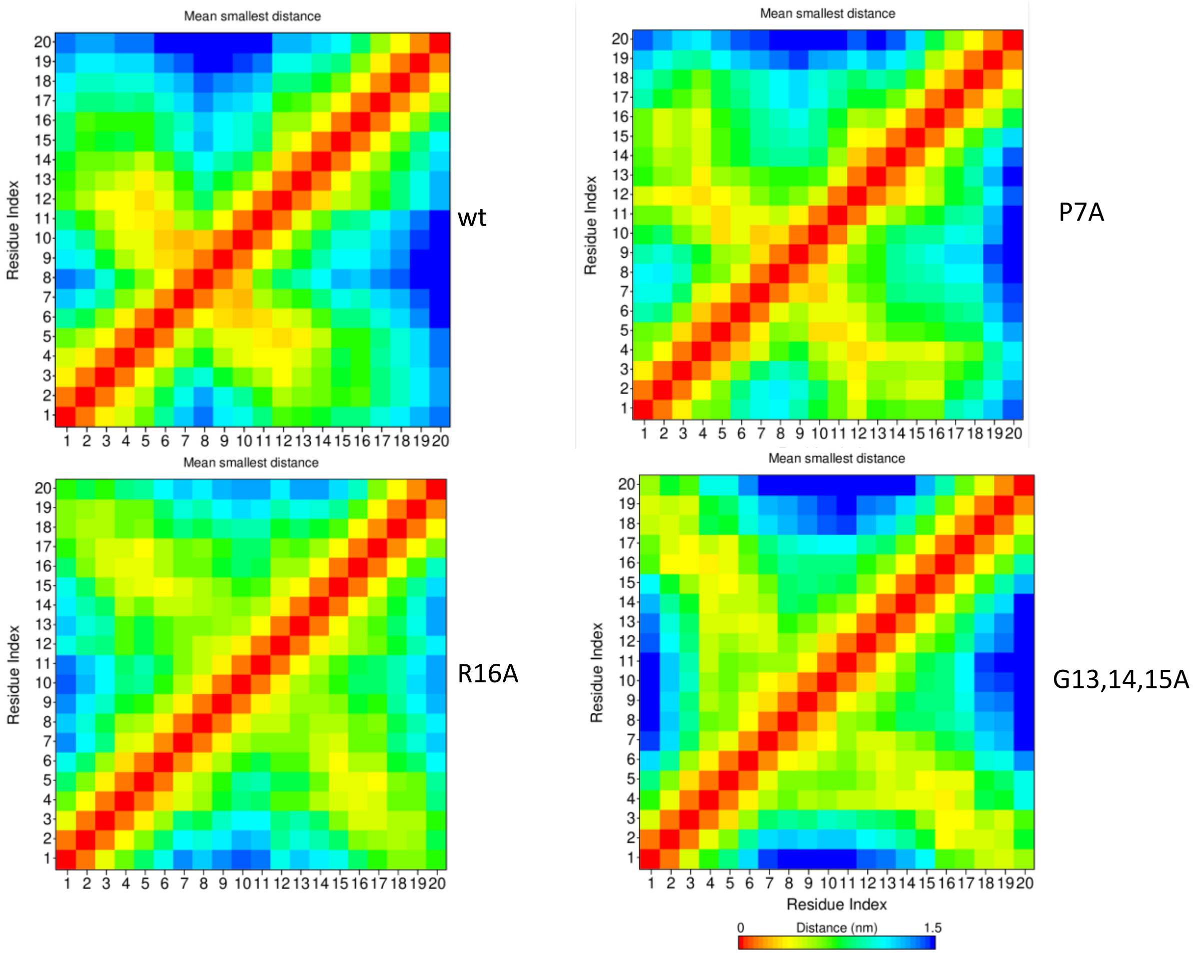

Supplementary Figure S4: MD simulations of SCOOP10#2 mutants.

(**A**) DSSP secondary structures calculated along the trajectories.

(**B**) Contact maps of selected mutants.

Supplementary Figure S5: Representative structures found in MD simulations for SCOOP10#2 and its mutants.

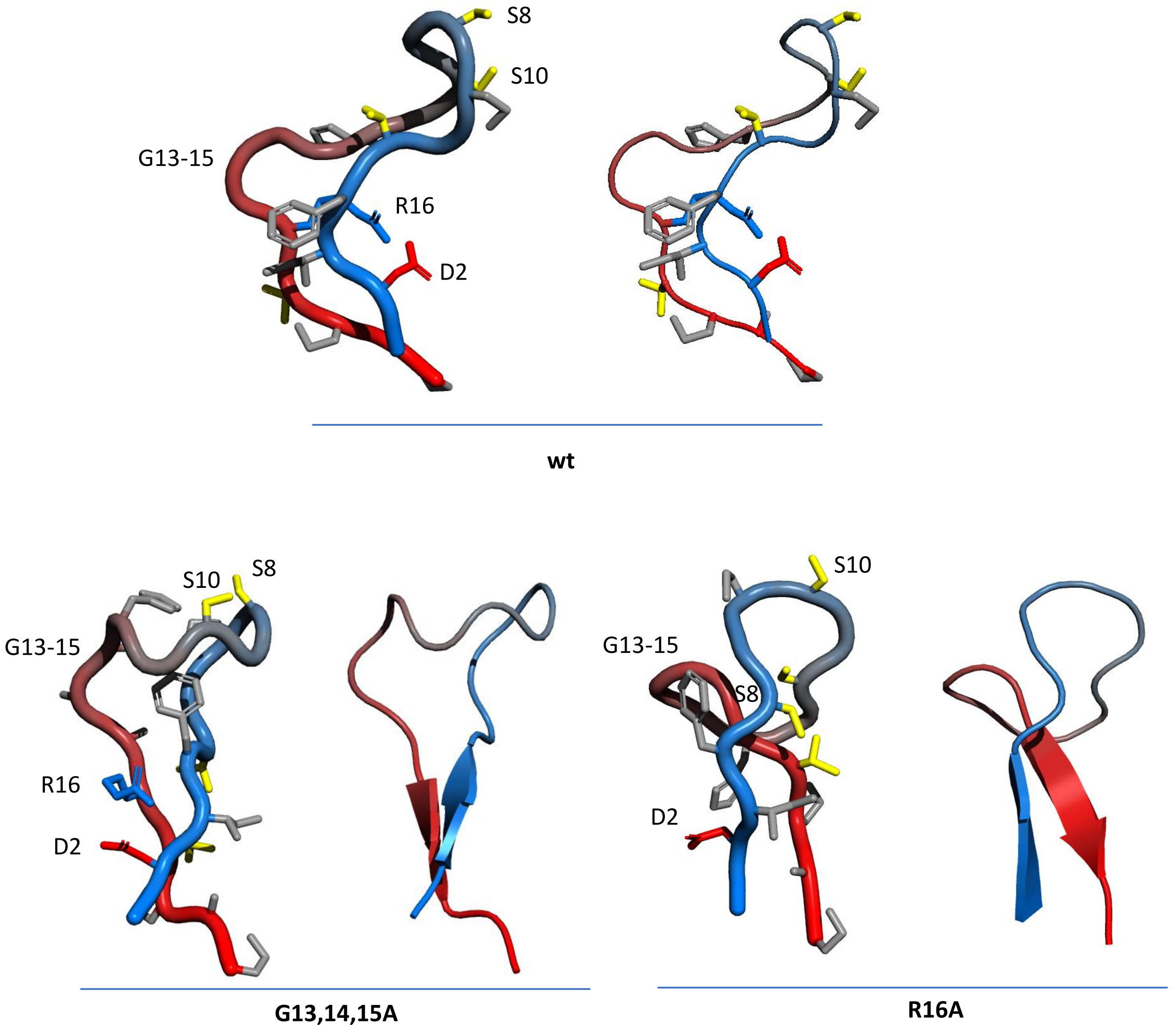

Supplementary Figure S5: Representative structures found in MD simulations for SCOOP10#2 and its mutants.

For each structure the tube (left) and ribbon (right) renderings are shown.

Supplementary Figure S6: Structural behaviour of SCOOP10#1 in solution as monitored by NMR.

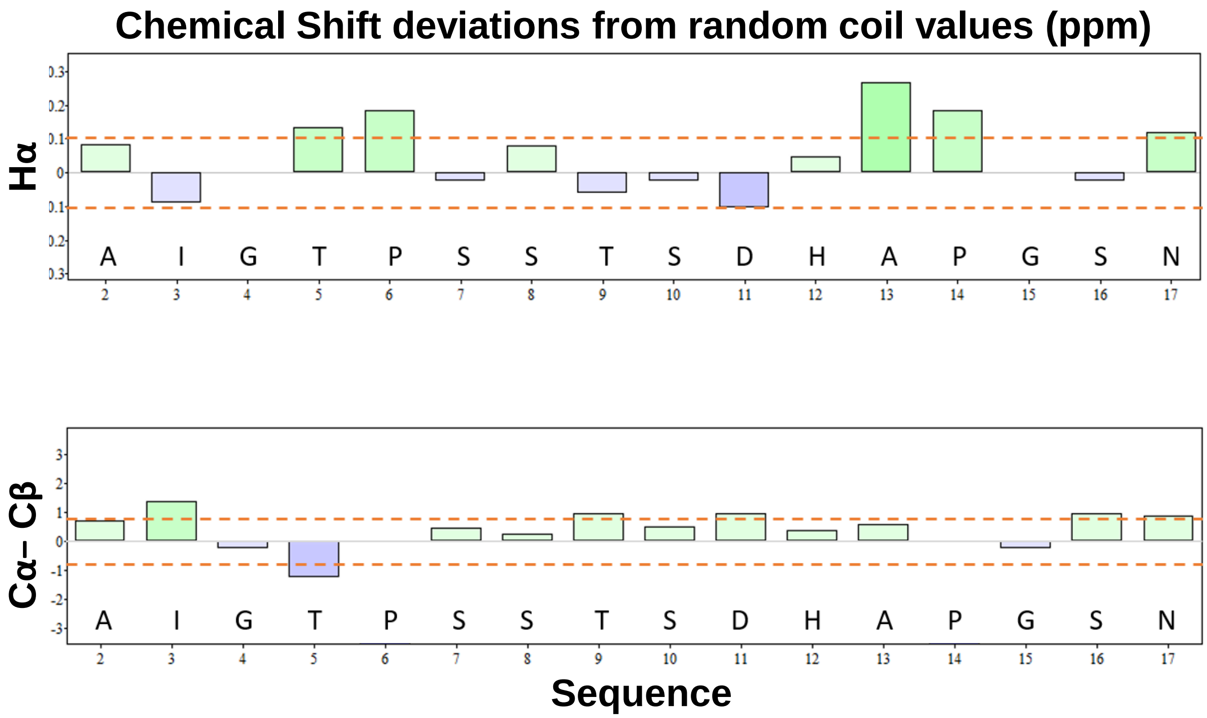

Supplementary Figure S6: Structural behaviour of SCOOP10#1 in solution as monitored by NMR.

Chemical shifts deviations from random coil values of Hα protons and of the difference between Cα and Cβ carbons suggest the absence of a well definite structure for SCOOP10#1. Deviations for glycine Hα atoms were intentionally omitted.

Supplementary Figure S7: Secondary structures and intramolecular interactions found in MD simulations of SCOOP10#1.

SCOOP10#1

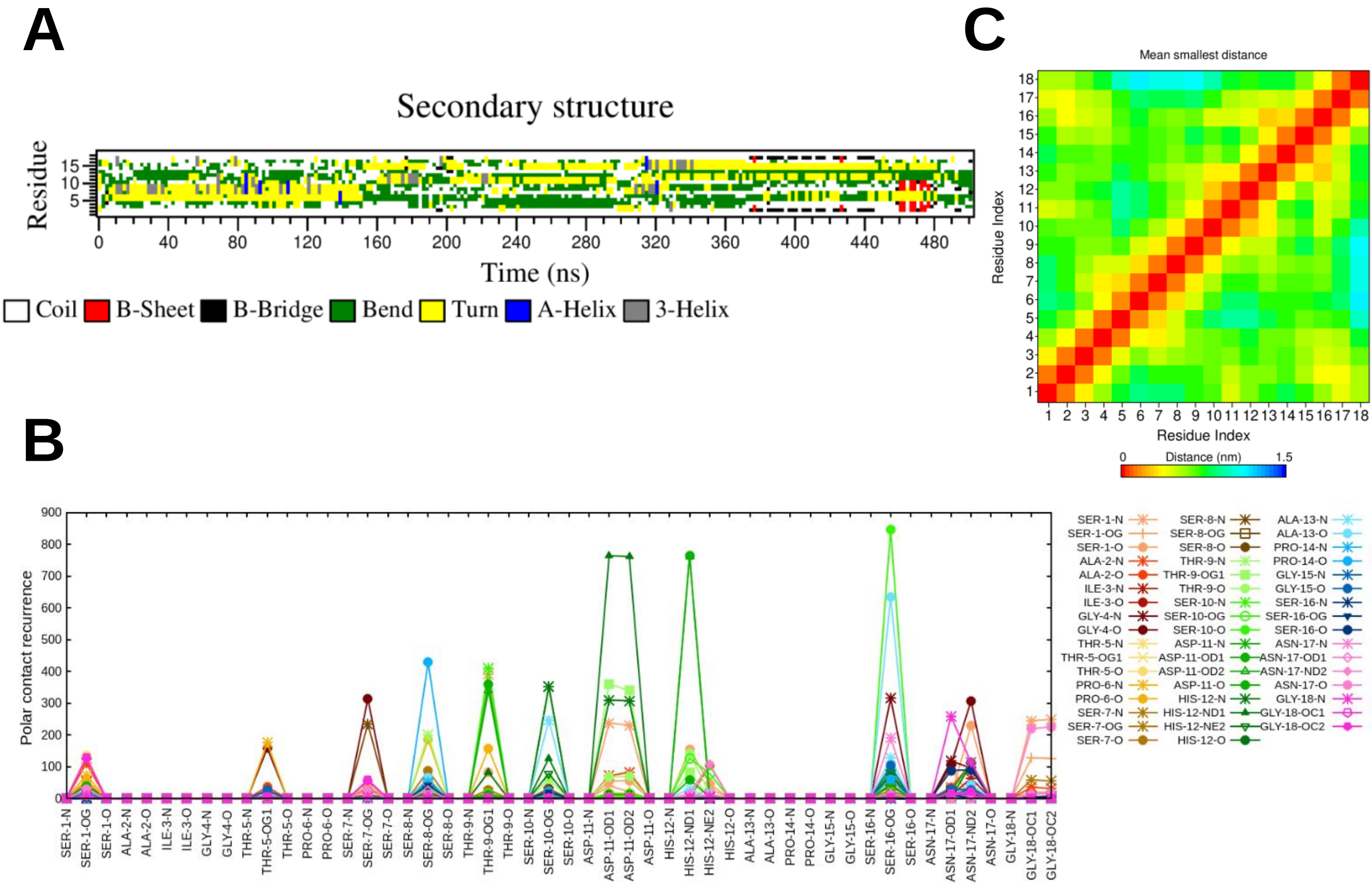

Supplementary Figure S7: Secondary structures and intramolecular interactions found in MD simulations of SCOOP10#1.

(**A**) DSSP secondary structures calculated along molecular dynamics (MD) simulation of hydroxylated SCOOP10#1 in solution. (**B**) Occurrence of intramolecular polar atom contacts (H-bonds and salt bridges) in SCOOP10#1 calculated along MD simulation trajectories. (**C**) Contact map.
