# Supplementary material for: The PROSCOOP10 Gene Encodes Two Extracellular Hydroxylated Peptides and Impacts Flowering Time in Arabidopsis
Source: Plants (Basel). 2022 Dec 16;11(24):3554. doi: 10.3390/plants11243554 (PMC9784617; doi:10.3390/plants11243554)
Supplement: Supplementary file 1 [file plants-11-03554-s001.zip › plants-2029070-supplementary/Supplementary_Data/Supplementary Tables S2-S7.pdf]

Supplementary Table S2: ^{1}H and ^{13}C NMR assignment of SCOOP10#2* peptide 1 mM in 50 mM phosphate buffer (10% D_2O), pH 6.6, 278K

	Gly1	Asp2	Ile3	Phe4	Thr5	Gly6	Pro7	Ser8
**^{1}H**	NH=x	NH = 8.721	NH= 8.361	NH = 8.635	NH = 8.256	NH = 7.665	NH= –	NH = 8.684
	α_2 = 3.862	α = 4.682	α = 4.151	α = 4.760	α = 4.340	α_2 = 3.995	α = 4.494	α = 4.458
	α_3 = 3.838	β_2 = 2.603	β = 1.797	β_2 = 3.112	β = 4.220	α_3 = 3.995	β_2 = 2.342	β_2 = 3.907
		β_3 = 2.558	γ_{12} = 1.355	β_3 = 3.112	γ = 1.179		β_3 = 2.001	β_3 = 3.907
			γ_{13} = 1.120	δ = 7.279			δ_2 = 3.689	
			γ_2 = 0.822	ε = 7.304			δ_3 = 3.648	
			δ = 0.833	ζ= 7.275			γ_2 = 2.067	
							γ_3 = 2.053	
**^{13}C**	α = 43.471	α = 54.668	α = 61.228	α = 57.818	α = 61.739	α = 45.456	α = 63.764	α = 58.772
		β = 41.901	β = 38.959	β = 39.768	β = 69.934		β = 32.340	β = 64.118
			γ_{12} = 27.328	δ = 132.023	γ = 21.545		δ = 49.951	
			γ_{13} = 27.328	ε = 131.891			γ = 27.454	
			γ_2 = 17.499	ζ= 131.322				
			δ = 13.107					

	Gly9	Ser10	Gly11	His12	Gly13	Gly14	Gly15	Arg16
**^{1}H**	NH = 8.564	NH = 8.415	NH=8.620	NH= 8.460	NH=8.748	NH= 8.527	NH=8.478	NH= 8.361
	α_2 = 4.018	α = 4.457	α_2 = 3.928	α = 4.722	α_2 = 3.995	α_2 = 3.995	α_2 = 3.995	α = 4.415
	α_3 = 3.995	β_2 = 3.891	α_3 = 3.992	β_2 = 3.271	α_3 = 3.995	α_3 = 3.995	α_3 = 3.995	β_2 = 1.853
		β_3 = 3.907		β_3 = 3.271				β_3 = 1.760
				δ = 7.151				δ_2 = 3.191
				ε = 8.274				δ_3 = 3.191
								γ= 1.615
**^{13}C**	α = 45.456	α = 58.772	α = 45.382	α = 55.929	α = 45.456	α = 45.456	α = 45.456	α = 56.023
		β = 64.118		β = 30.007				β = 31.101
				δ = 30.219				δ = 43.462
				ε = 137.435				γ = 27.188

	Thr17	Pro18	Ala19	Pro20
^{1}H	NH = 8.489	NH= –	NH= 8.533	NH= –
	α = 4.571	α = 4.399	α = 4.571	α = 4.240
	β = 4.130	$β_2$ = 2.290	β = 1.388	$β_2$ = 2.240
	γ = 1.269	$β_3$ = 1.967		$β_3$ = 1.908
		$δ_2$ = 3.890		$δ_2$ = 3.756
		$δ_3$ = 3.705		$δ_3$ = 3.659
		$γ_2$ = 2.021		$γ_2$ = 1.992
		$γ_3$ = 2.045		$γ_3$ = 1.992
^{13}C	α = 60.353	α = 63.299	α = 50.439	α = 64.755
	β = 69.894	β = 32.316	β = 18.114	β = 32.153
	γ = 21.575	δ = 51.358		δ = 50.240
		γ = 27.520		γ = 27.414

Supplementary Table S3: ^{1}H and ^{13}C NMR assignment of hydroxylated SCOOP10#2 peptide 0.5 mM in 50 mM phosphate buffer (10% D_2O), pH 6.6, 278K

	Gly1	Asp2	Ile3	Phe4	Thr5	Gly6	Hyp7	Ser8
**^{1}H**	NH=x<br>α_2 = 3.844<br>α_3 = 3.772	NH = 8.719<br>α = 4.673<br>β_2 = 2.655<br>β_3 = 2.542	NH= 8.368<br>α = 4.154<br>β = 1.797<br>γ_{12} = 1.374<br>γ_{13} = 1.13<br>γ_2 = 0.824<br>δ = 0.827	NH = 8.639<br>α = 4.748<br>β_2 = 3.113<br>β_3 = 3.084<br>δ = 7.265<br>ε = 7.317<br>ζ= 7.277	NH = 8.268<br>α = 4.349<br>β = 4.229<br>γ = 1.164	NH = 7.591<br>α_2 = 4.074<br>α_3 = 3.990	NH= –<br>α = 4.586<br>β_2 = 2.331<br>β_3 = 1.878<br>δ_2 = 3.840<br>δ_3 = 3.840<br>γ = 4.633	NH = 8.813<br>α = 4.459<br>β_2 = 3.927<br>β_3 = 3.908
**^{13}C**	α = 43.419	α = 54.473<br>β_2 = 41.269	α = 61.395<br>β = 38.959<br>γ_{12} = 27.367<br>γ_{13} = 27.367<br>γ_2 = 17.501<br>δ = 13.089	α = 57.961<br>β = 39.976<br>δ = 132.105<br>ε = 131.782<br>ζ= 130.103	α = 62.636<br>β = 69.934<br>γ = 21.558	α = 45.432	α = 62.081<br>β_2 = 31.151<br>β_3 = 31.151<br>δ = 57.504<br>γ = 72.723	α = 58.842<br>β = 63.909

	Gly9	Ser10	Gly11	His12	Gly13	Gly14	Gly15	Arg16
**^{1}H**	NH = 8.577<br>α_2 = 3.984<br>α_3 = 3.948	NH = 8.408<br>α = 4.462<br>β_2 = 3.857<br>β_3 = 3.846	NH=8.607<br>α_2 = 3.969<br>α_3 = 3.969	NH= 8.442<br>α = 4.719<br>β_2 = 3.290<br>β_3 = 3.113<br>δ = 7.151<br>ε = 8.274	NH=8.696<br>α_2 = 3.983<br>α_3 = 3.986	NH= 8.509<br>α_2 = 3.960<br>α_3 = 3.979	NH=8.468<br>α_2 = 3.968<br>α_3 = 3.976	NH= 8.362<br>α = 4.425<br>β_2 = 1.807<br>β_3 = 1.774<br>δ_2 = 3.191<br>δ_3 = 3.158<br>γ_2 = 1.598<br>γ_3 = 1.584
**^{13}C**	α = 45.493	α = 58.851<br>β = 63.899	α = 45.441	α = 55.911<br>β = 29.227<br>δ = 30.219<br>ε = 137.435	α = 45.470	α = 45.538	α = 45.424	α = 56.024<br>β_2 = 31.183<br>β_3 = 31.183<br>δ = 43.421<br>γ = 27.443

	Thr17	Hyp18	Ala19	Pro20
**^{1}H**	NH = 8.526	NH= –	NH= 8.638	NH= –
	α = 4.581	α = 4.530	α = 4.575	α = 4.232
	β = 4.123	β_2 = 2.338	β = 1.396	β_2 = 2.226
	γ = 1.257	β_3 = 1.876		β_3 = 1.897
		δ_2 = 3.840		δ_2 = 3.746
		δ_3 = 3.840		δ_3 = 3.630
		γ = 4.595		γ = 1.981
**^{13}C**	α = 60.217	α = 61.953	α = 50.668	α = 63.844
	β = 69.894	β_2 = 31.164	β = 18.025	β_2 = 32.162
	γ = 21.558	β_3 = 31.164		β_3 = 32.162
		δ = 57.504		δ = 50.288
		γ = 72.751		γ = 27.503

Supplementary Table S4: ^{1}H and ^{13}C NMR assignment of SCOOP10#2* peptide 0.5 mM in DMSO, 298K

	Gly1	Asp2	Ile3	Phe4	Thr5	Gly6	Pro7	Ser8
**^{1}H**	NH=x	NH = 8.643	NH= 7.933	NH = 8.031	NH = 7.981	NH = 7.833	NH= –	NH = 8.156
	α_2 = 3.590	α = 4.716	α = 4.134	α = 4.730	α = 4.302	NH^{cis} = 7.767	α = 4.446	NH^{cis} = 8.367
	α_3 = 3.590	β_2 = 2.627	β = 1.682	β_2= 3.106	β = 4.053	α_2 = 4.066	β_2 = 2.068	α = 4.255
		β_3 = 2.503	γ_{12} = 1.338	β_3= 2.832	γ = 1.064	α_2^{cis} = 3.983	β_3 = 1.909	α^{cis} = 4.395
			γ_{13} = 1.338	δ = 7.292		α_3 = 3.865	δ_2 = 3.557	β_2 = 3.619
			γ_2 = 0.722	ε = 7.272		α_3^{cis} = 3.514	δ_3 = 3.530	β_2^{cis} = 3.650
			δ = 0.772	ζ= 7.202			γ_2 = 1.906	β_3 = 3.619
							γ_3 = 1.906	β_3^{cis} = 3.650
**^{13}C**	α = 40.601	α = 50.056	α = 57.815	α = 54.050	α = 58.609	α = 42.266	α = 60.196	α = 56.020
		β = 37.103	β = 37.347	β = 37.981	β = 67.175		β = 29.888	β = 62.185
			γ_{12} = 24.712	δ = 129.876	γ = 19.966		δ = 46.623	
			γ_{13} = 24.712	ε = 128.645			γ = 24.953	
			γ_2 = 15.909	ζ= 126.871				
			δ = 11.822					

	Gly9	Ser10	Gly11	His12	Gly13	Gly14	Gly15	Arg16
**^{1}H**	NH= 8.066	NH = 8.081	NH= 8.263	NH= 8.111	NH=8.320	NH= 8.177	NH= 8.175	NH= 8.070
	NH^{cis}= 8.226	NH^{cis} = 8.060	α_2 = 3.766	α = 4.646	α_2 = 3.859	α_2 = 3.822	α_2 = 3.821	α = 4.437
	α_2 = 3.855	α = 4.314	α_3 = 3.766	β_2 = 3.154	α_3 = 3.796	α_3 = 3.809	α_3 = 3.766	β_2 = 1.696
	α_3 = 3.855	α^{cis} = 4.319		β_3 = 3.001				β_3 = 1.518
	α^{cis} = 3.847	β_2 = 3.605		δ = –				δ_2 = 3.111
		β_2^{cis} = 3.634		ε = –				δ_3 = 3.111
		β_3 = 3.605						γ = 1.494

		β_3^{cis} = 3.634						
**^{13}C**	α = 42.481	α = 55.927	α = 42.152	α = 52.107	α = 42.649	α = 42.528	α = 42.590	α = 52.183
		β = 62.151		β = 27.537				β = 30.091
				δ = –				δ = 41.010
				ε = –				γ = 25.506

	**Thr17**	**Pro18**	**Ala19**	**Pro20**				
**^{1}H**	NH = 8.047	NH= –	NH= 8.061	NH= –				
	α = 4.385	α = 4.368	α = 4.524	α = 4.255				
	β = 3.878	β_2 = 2.044	β = 1.211	β_2 = 2.178				
	γ = 1.167	β_3 = 1.855		β_3 = 1.863				
		δ_2 = 3.778		δ_2 = 3.640				
		δ_3 = 3.667		δ_3 = 3.526				
		γ_2 = 1.849		γ_2 = 1.939				
		γ_3 = 1.889		γ_3 = 1.939				
**^{13}C**	α = 57.439	α = 59.793	α = 46.810	α = 59.125				
	β = 67.594	β = 29.622	β = 17.386	β = 29.192				
	γ = 20.016	δ = 48.017		δ = 47.019				
		γ = 25.212		γ = 25.193				

Supplementary Table S5: ^{1}H and ^{13}C NMR assignment of hydroxylated SCOOP10#2 peptide 0.5 mM in DMSO, 298K

	Gly1	Asp2	Ile3	Phe4	Thr5	Gly6	Hyp7	Ser8
**^{1}H**	NH=x	NH = 8.636	NH= 7.933	NH = 8.079	NH = 8.012	NH = 7.858	NH= –	NH = 8.300
	α_2 = 3.596	α = 4.708	α = 4.134	α = 4.719	α = 4.276	NH^{cis} = 7.751	α = 4.447	NH^{cis} = 8.555
	α_3 = 3.596	β_2 = 2.631	β = 1.682	β_2= 3.111	β = 4.051	α_2 = 4.062	β_2 = 2.062	α = 4.254
		β_3 = 2.603	γ_{12} = 1.338	β_3= 2.857	γ = 1.070	α_2^{cis} = 3.965	β_3 = 1.952	α^{cis} = 4.401
			γ_{13} = 1.338	δ = 7.425		α_3 = 3.841	δ_2 = 3.647	β_2 = 3.656
			γ_2 = 0.722	ε = 7.263		α_3^{cis} = 3.553	δ_3 = 3.405	β_2^{cis} = 3.669
			δ = 0.772	ζ= 7.203			γ_2 = 4.387	β_3 = 3.656
							γ_3 = 4.387	β_3^{cis} = 3.669
**^{13}C**	α = 40.912	α = 50.321	α = 58.020	α = 54.291	α = 58.855	α = 42.658	α = 59.182	α = 56.177
		β = 41.769	β = 39.354	β = 40.984	β = 67.219		β = 38.149	β = 62.268
			γ_{12} = –	δ = 129.874	γ = 20.104		δ = 54.680	
			γ_{13} = –	ε = 128.743			γ = 69.504	
			γ_2 = 15.909	ζ= 126.907				
			δ = 11.822					

	Gly9	Ser10	Gly11	His12	Gly13	Gly14	Gly15	Arg16
**^{1}H**	NH= 8.086	NH = 8.038	NH= 8.263	NH= 7.992	NH=8.377	NH= 8.177	NH= 8.175	NH= 8.039
	NH^{cis}= 8.226	NH^{cis} = 8.087	α_2 = 3.766	α = 4.473	α_2 = 3.825	α_2 = 3.822	α_2 = 3.824	α = 4.426
	α_2 = 3.809	α = 4.319	α_3 = 3.766	β_2 = 3.000	α_3 = 3.728	α_3 = 3.818	α_3 = 3.766	β_2 = 1.739
	α_3 = 3.809	α^{cis} = 4.328		β_3 = 2.903				β_3 = 1.571
	α^{cis} = 3.847	β_2 = 3.628		δ = –				δ_2 = –
		β_2^{cis} = 3.634		ε = –				δ_3 = –
		β_3 = 3.628						γ = 1.528

		β_3^{cis} = 3.634						
**^{13}C**	α = 42.954	α = 56.029	α = 42.152	α = 53.751	α = 42.757	α = 42.899	α = 42.685	α = 52.341
		β = 62.245		β = –				β = 29.990
				δ = –				δ = –
				ε = –				γ = 25.657

	**Thr17**	**Hyp18**	**Ala19**	**Pro20**				
**^{1}H**	NH = 7.963	NH= –	NH= 8.155	NH= –				
	α = 4.422	α = 4.433	α = 4.518	α = 4.236				
	β = 3.888	β_2 = 2.034	β = 1.211	β_2 = 2.178				
	γ = 1.132	β_3 = 1.875		β_3 = 1.838				
		δ_2 = 3.748		δ_2 = 3.640				
		δ_3 = 3.619		δ_3 = 3.526				
		γ_2 = 4.338		γ_2 = 1.951				
		γ_3 = 4.338		γ_3 = 1.918				
**^{13}C**	α = 57.408	α = 58.988	α = 46.810	α = 59.125				
	β = 67.594	β = 38.425	β = 17.386	β = 29.255				
	γ = 19.844	δ = 56.170		δ = 47.019				
		γ = 69.252		γ = 25.224				

Supplementary Table S6: ^{1}H and ^{13}C NMR assignment of hydroxylated SCOOP10#1 peptide 0.5 mM in 50 mM phosphate buffer (10% D_2O), pH 6.6, 278K

	Ser1	Ala2	Ile3	Gly4	Thr5	Hyp6	Ser7	Ser8	Thr9
**^{1}H**	NH = x	NH = 8.812	NH= 8.453	NH= 8.657	NH = 8.234	NH= –	NH = 8.769	NH = 8.598	NH = 8.369
	α = 4.108	α = 4.416	α = 4.126	α_2 = 3.992	α = 4.605	α = 4.602	α = 4.475	α = 4.574	α = 4.414
	β_2 = 3.978	β = 1.385	β = 1.843	α_3 = 3.985	β = 4.136	β_2 = 2.384	β_2 = 3.934	β_2 = 3.938	β = 4.306
	β_3 = 3.936		γ_{12} = 1.247		γ = 1.250	β_3 = 2.081	β_3 = 3.883	β_3 = 3.885	γ = 1.211
			γ_{13} = 1.190			δ_2 = 3.972			
			γ_2 = 0.934			δ_3 = 3.867			
			δ = 0.898			γ_2 = 4.597			
						γ_3 = 4.630			
**^{13}C**	α = 57.440	α = 52.496	α = 61.647	α = 45.030	α = 60.138	α = 62.345	α = 58.630	α = 58.526	α = 62.104
	β = 63.273	β = 19.456	β = 38.970		β = 69.950	β_2 = 39.910	β = 63.936	β = 64.042	β = 69.730
			γ_{12} = 27.05		γ = 21.502	β_3 = 39.910			γ = 21.706
			γ_{13} = 27.79			δ = 58.928			
			γ_2 = 17.60			γ = 72.850			
			δ = 12.99						

	Ser10	Asp11	His12	Ala13	Hyp14	Gly15	Ser16	Asn17	Gly18
**^{1}H**	NH = 8.361	NH= 8.357	NH= 8.375	NH= 8.591	NH= --	NH= 8.870	NH = 8.299	NH= 8.734	NH= 8.076
	α = 4.445	α = 4.544	α = 4.668	α = 4.590	α = 4.595	α_2 = 4.015	α = 4.473	α = 4.802	α_2 = 3.767
	β_2 = 3.877	β = 2.624	β_2 = 3.246	β = 1.355	β_2 = 2.375	α_3 = 4.031	β_2 = 3.882	β_2 = 2.876	α_3 = 3.707
	β_3 = 3.821		β_3 = 3.141		β_3 = 2.082		β_3 = 3.936	β_3 = 2.736	
			δ = 7.259		δ_2 = 3.930				
			ε = 8.559		δ_3 = 3.827				
					γ_2 = 4.633				
					γ_3 = 4.628				
**^{13}C**	α = 58.630	α = 54.619	α = 55.067	α = 50.838	α = 62.345	α = 45.050	α = 58.630	α = 53.411	α = 46.246

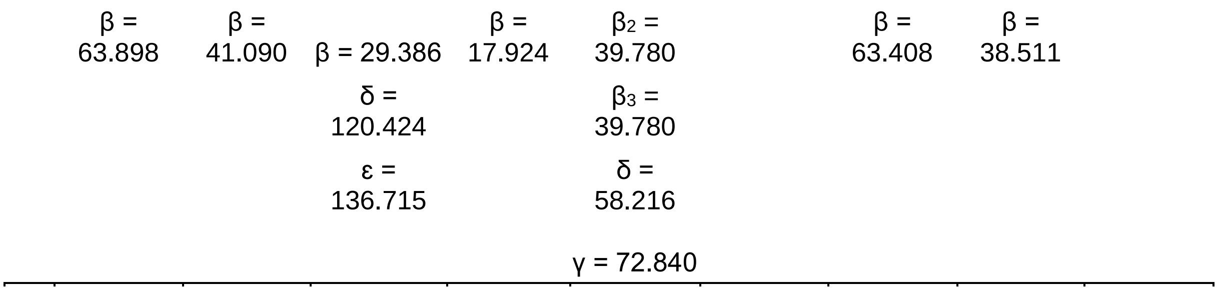
β = 63.898
β = 41.090
β = 29.386
δ = 120.424
ε = 136.715
β = 17.924
β₂ = 39.780
β₃ = 39.780
δ = 58.216
γ = 72.840
β = 63.408
β = 38.511

Supplementary Table S7: ^{1}H and ^{13}C NMR assignment of hydroxylated SCOOP10#1 peptide 0.5 mM in DMSO, 298K

	Ser1	Ala2	Ile3	Gly4	Thr5	Hyp6	Ser7	Ser8	Thr9
**^{1}H**	NH = x	NH = 8.638	NH= 7.961	NH= 8.200	NH = 7.939	NH= –	NH = 8.111	NH = 7.958	NH=7.790
	α = 3.881	α = 4.464	α = 4.214	α$_2$ = 3.888	α = 4.502	α = 4.48	α = 4.343	α = 4.393	α = 4.335
	β$_2$ = 3.773	β = 1.273	β = 1.745	α$_3$ = 3.758	β = 3.864	β$_2$ = 2.058	β$_2$ = 3.639	β$_2$ = 3.716	β = 4.069
	β$_3$ = 3.646		γ$_{12}$ = 1.468		γ = 1.119	β$_3$ = 1.959	β$_3$ = 3.636	β$_3$ = 3.623	γ = 1.066
			γ$_{13}$ = 1.114			δ$_2$ = 3.753			
			γ$_2$ = 0.872			δ$_3$ = 3.634			
			δ = 0.846			γ$_2$ = 4.349			
						γ$_3$ = 4.342			
**^{13}C**	α = 54.989	α = 49.093	α = 57.625	α = 42.391	α = 57.065	α = 59.235	α = 55.784	α = 55.717	α = 58.633
	β = 61.246	β = 18.821	β = 37.487		β = 67.844	β = 38.268	β = 62.206	β = 62.239	β = 67.241
			[illegible]$_2$ = 24.976		γ = 19.925	δ = 56.352			γ = 20.049
			[illegible]$_3$ = 24.976			γ = 69.326			
			γ$_2$ = 16.046						
			δ = 11.860						

	Ser10	Asp11	His12	Ala13	Hyp14	Gly15	Ser16	Asn17	Gly18
**^{1}H**	NH = 7.928	NH=7.950	NH= 8.292	NH= 8.228	NH= –	NH=8.281	NH = 7.886	NH= 8.312	NH=8.041
	α = 4.363	α = 4.579	α = 4.585	α = 4.495	α = 4.420	α$_2$ = 3.819	α = 4.375	α = 4.642	α$_2$ = 3.760
	β$_2$ = 3.646	β$_2$ = 3.085	β$_2$ = 2.725	β = 1.244	β$_2$ = 2.077	α$_3$ = 3.718	β$_2$ = 3.647	β$_2$ = 2.603	α$_3$ = 3.759
	β$_3$ = 3.604	β$_3$ = 2.983	β$_3$ = 2.539		β$_3$ = 1.946		β$_3$ = 3.590	β$_3$ = 2.474	
			δ = –		δ$_2$ = 3.683				
			ε = –		δ$_3$ = 3.545				
					γ$_2$ = 4.412				
					γ$_3$ = 4.411				

**^{13}C**	α = 55.481	α = 50.335	α = 52.083	α = 47.475	α = 59.634	α = 42.601	α = 55.706	α = 50.328	α = 41.512
	β = 62.410	β = –	β = 36.252	β = 16.939	β = 38.269		β = 62.481	β = 37.514	
			δ = –		δ = 55.594				
			ε = –		γ = 69.444				
